# Respiratory Syncytial Virus (RSV): A Comprehensive Overview From Basic Biology to Clinical Prevention and Control

**DOI:** 10.1002/med.70025

**Published:** 2025-11-19

**Authors:** Jie Shi, Xiya Huang, Chunjun Ye, Yishan Lu, Yanyan Liu, Yuquan Wei, Xiawei Wei

**Affiliations:** ^1^ Laboratory of Aging Research and Cancer Drug Target, State Key Laboratory of Biotherapy and Cancer Center, National Clinical Research Center for Geriatrics, West China Hospital, Sichuan University Chengdu Sichuan People's Republic of China

**Keywords:** basic biology, clinical control, clinical prevention, RSV, RSV vaccines

## Abstract

Respiratory syncytial virus (RSV) is a common virus that causes respiratory infections, posing a serious threat, particularly to infants, the elderly, and individuals with compromised immune systems. As the leading cause of lower respiratory tract infections (LRTIs) in infants, RSV is responsible for millions of cases worldwide each year. Its incidence rises significantly during the winter influenza season. Despite decades of research, no effective vaccine exists, and antiviral treatment options remain limited, presenting a major challenge to global public health. With the advancement of emerging technologies, researchers have made significant progress in understanding the pathological and biological characteristics of RSV, the mechanisms of immune response, and its long‐term health impacts. This review aims to provide a comprehensive overview of the basic biological characteristics, epidemiology, clinical manifestations, and diagnostic and therapeutic strategies of RSV and to explore preventive measures and future research directions, offering the latest scientific evidence for RSV prevention and control.

**Abbreviations**
Ad26: adenovirus serotype 26
ADCC: antibody‐dependent cellular cytotoxicity
AHR: airway hyperreactivity
ALRI: acute lower respiratory tract infection
AM: alveolar macrophages
APC: antigen‐presenting cell
ASC: antibody‐secreting cell
BAFF: B‐cell activating factor of the TNF family
BALF: bronchoalveolar lavage fluid
BHR: bronchial hyperresponsiveness
BLP: bacterium‐like‐particle
COPD: chronic obstructive pulmonary disease
CTL: cytotoxic T lymphocyte
DAMP: damage‐associated molecular pattern
DLL3: delta‐like 3
ECM: extracellular matrix
ECP: eosinophil cationic protein
EDN: eosinophil‐derived neurotoxin
EETs: eosinophil extracellular traps
EGFR: epidermal growth factor receptor
GC: germinal center
hBEC: human bronchial epithelial cell
HLA: human leukocyte antigen
HMGB1: high‐mobility group box‐1
HSCT: hematopoietic stem cell transplantation
ICAM‐1: intercellular adhesion molecule‐1
IFN: interferon
IGF‐1R: insulin‐like growth factor‐1 receptor
ILC2: type 2 innate lymphoid cell
LAV: live‐attenuated vaccine
LNP: lipid nanoparticle
LRTD: lower respiratory tract disease
LRTI: lower respiratory tract infection
mAb: monoclonal antibody
MBMA: model‐based meta‐analysis
MBP: major basic protein
MHC: major histocompatibility complex
MLKL: mixed lineage kinase domain‐like
MMP‐12: matrix metalloproteinase‐12
MPO: myeloperoxidase
nBreg cell: regulatory B lymphocyte
NCL: nucleolin
NET: neutrophil extracellular trap
ORF: open reading frame
PAMP: pathogen‐associated molecular pattern
PIV5: parainfluenza virus 5
RIPK: receptor‐interacting protein kinase
RNP: ribonucleoprotein
ROS: reactive oxygen species
RSV: respiratory syncytial virus
RSV‐LRTD: RSV lower respiratory tract disease
SeV: sendai virus
Tfh: follicular helper T
TGF: transforming growth factor
TLR: toll‐like receptor
TNF: tumor necrosis factor
Treg: regulatory T cell
TRM: Tissue‐resident memory T
VAERD: vaccine‐associated enhanced respiratory disease
VAP: ventilator‐associated pneumonia
VITT: vaccine‐induced immune thrombotic thrombocytopenia
VLP: virus‐like particle
WHO: World Health Organization

## 1 Introduction

Respiratory syncytial virus (RSV) is a highly contagious virus and one of the leading causes of lower respiratory tract infections (LRTIs) in infants [1]. Each year, RSV causes millions of cases of illness in infants and children worldwide, with a significant increase in incidence during the winter influenza season. Although RSV infection typically presents with mild upper respiratory symptoms, it can lead to severe lower respiratory tract diseases (LRTDs), such as bronchiolitis and pneumonia. It may even be life‐threatening in high‐risk populations, including premature infants, the elderly, immunocompromised individuals, and patients with chronic lung or heart disease [2, 3].

Although RSV was discovered decades ago, effective prevention and control measures have remained limited for a long time. Current antiviral treatment strategies primarily focus on supportive care, such as oxygen therapy, ventilation, and fluid management [4, 5]. In recent years, significant progress has been made in addressing this challenge. We conducted a literature search in the PubMed database and found that in 2023, the first RSV vaccines were approved, marking a breakthrough in preventing and controlling this virus. Among them, Arexvy (GSK), Abrysvo (Pfizer), and mRESVIA (Moderna) are three newly approved vaccines primarily designed to protect adults aged 60 and older from LRTDs caused by RSV infection, aiming to reduce RSV‐related severe illness and mortality. Notably, Abrysvo is also approved for maternal administration during pregnancy to provide passive immunity to infants [6, 7]. However, despite the introduction of these vaccines marking a significant advancement in RSV prevention and control, several challenges remain, including immune tolerance, the limitations of local immune responses, and individual differences in immune responses. With repeated infections, the host immune system may gradually develop a tolerance to RSV, leading to a weakened immune response. Moreover, current vaccines and therapeutic strategies primarily focus on systemic immunity while overlooking the importance of mucosal immunity. The insufficiency of local immune responses makes it difficult for respiratory epithelial cells to prevent RSV invasion. Additionally, immune responses vary significantly among different populations, such as neonates, the elderly, and immunocompromised individuals, requiring prevention strategies to be tailored to the characteristics of each group. More importantly, RSV infection is closely associated with the development of chronic respiratory diseases, such as asthma, further increasing the health burden and socioeconomic costs [8]. Therefore, in‐depth research into the pathological characteristics and immune mechanisms of RSV, along with the development of vaccines capable of inducing both systemic and local immune responses, will provide a more comprehensive scientific basis for the integrated prevention and control of RSV.

In recent years, significant progress has been made in the study of the pathobiology, immunological mechanisms, and epidemiological characteristics of RSV, laying an important foundation for the development of novel vaccines and therapeutic strategies. A deeper understanding of RSV transmission patterns, pathogenic mechanisms, and host immune responses is crucial for developing more effective prevention and control strategies. This review systematically summarizes the basic biological characteristics, epidemiology, clinical manifestations, and diagnostic and therapeutic methods of RSV and evaluates current preventive measures, particularly the newly approved vaccines, and future research directions, providing scientific guidance and important references for comprehensive RSV prevention and control.

## 2 The Basic Biological Characteristics of RSV

**Figure 1:**
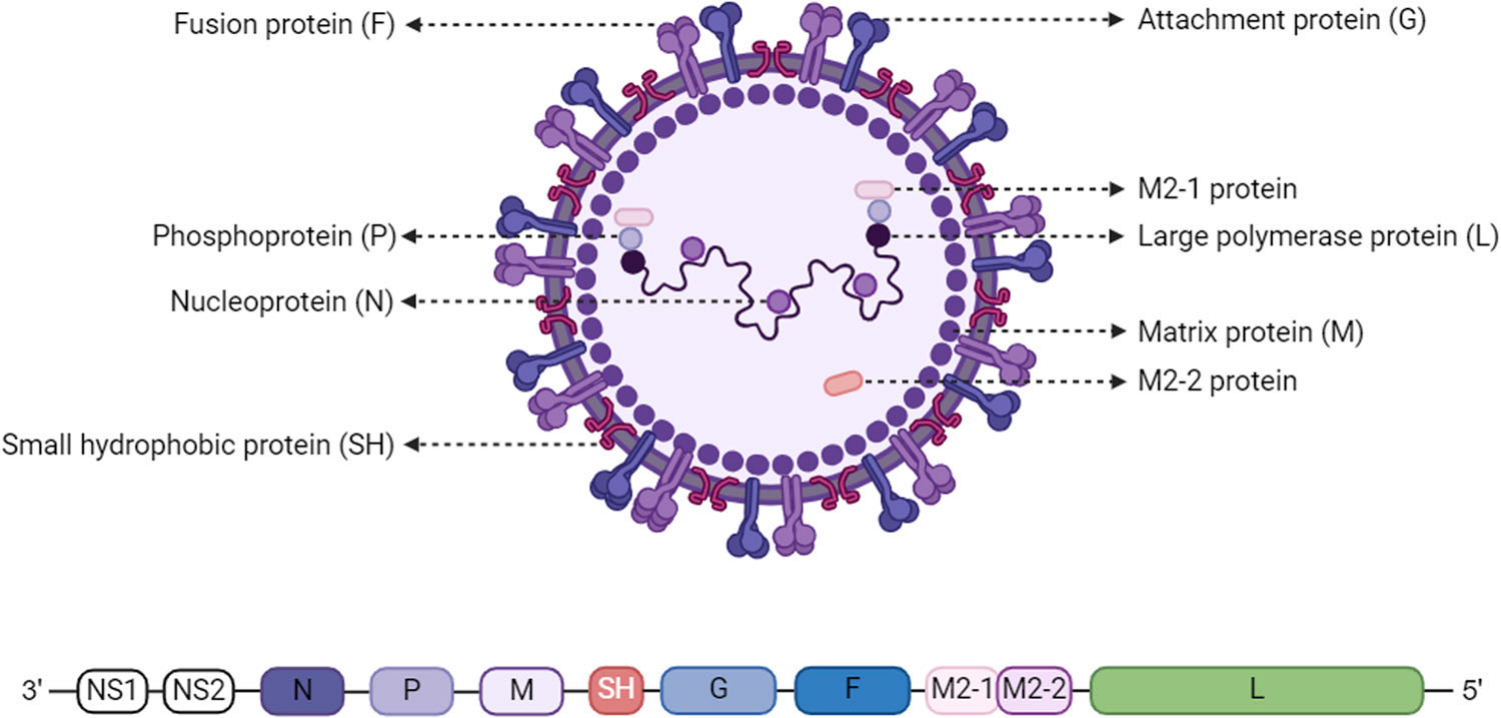
Structure and genome of RSV virus. [Color figure can be viewed at wileyonlinelibrary.com]

RSV is a pleomorphic, enveloped, single‐stranded negative‐sense RNA virus belonging to the Paramyxoviridae family. Its genome is approximately 15.2 kb in length and is transcribed through a sequential stop‐start mechanism, producing 10 mRNAs that encode 11 proteins. This is due to two overlapping open reading frames (ORFs) in the M2 mRNA, which produce two distinct matrix proteins, matrix protein 2‐1 (M2‐1) and matrix protein 2‐2 (M2‐2) [9] (Figure 1). Among the 11 proteins, nonstructural proteins 1 (NS1) and 2 (NS2) are primarily involved in the immune evasion of the virus [10], the small hydrophobic protein (SH) can inhibit host cell apoptosis [11]; the fusion protein (F) and attachment protein (G) are two major surface glycoproteins that mediate the attachment and fusion of the virus with host cells [12, 13], the nucleoprotein (N), large polymerase protein (L), phosphoprotein (P), matrix protein (M), M2‐1 protein, and M2‐2 protein are mainly responsible for the replication and transcription of the viral RNA [14, 15, 16]. The synergistic action of these proteins enables RSV to effectively infect host cells, replicate its genome, and evade immune system clearance. More importantly, RSV spreads through droplets or direct contact with secretions from infected individuals, making it highly contagious and capable of rapidly spreading within populations. Therefore, preventing and controlling RSV is crucial.

RSV is typically classified into two major antigenic subtypes: type A (RSV A) and type B (RSV B). These two subtypes are classified based on antigenic differences in their surface glycoproteins, with variations in the G protein being a key factor in distinguishing between the two subtypes. The G protein exhibits high antigenic variability in both RSV subtypes, primarily concentrated in nonsynonymous mutations and changes in glycosylation sites in the C‐terminal region of the G protein, leading to significant differences in antigenicity between the two subtypes [17, 18, 19]. Due to the high variability of the G protein, RSV constantly alters its antigenic surface structure, making it difficult for the host's immune system to recognize and produce effective neutralizing antibodies, helping the virus evade immune surveillance. As a result, the host may remain susceptible to a different subtype even after previously being infected with one subtype [19, 20]. As RSV continues to evolve globally, multiple genotypes have emerged over the years. RSV A strains can be classified into 14 genotypes (GA1‐GA7, NA1‐4, SAA1‐2, and ON1), while RSV B strains primarily consist of 23 genotypes (GB1–GB4, SAB1–SAB4, URU1‐2, BA1–BA12, and THB) [21]. The most commonly identified RSV A genotype is NA1, while the predominant RSV B genotype is BA [22]. Different subtypes and genotypes can coexist during each RSV epidemic season, leading to variations in disease severity. A recent study indicated that different RSV subtypes exhibit distinct clinical manifestations. Patients positive for RSV A are more likely to develop bronchiolitis, dyspnea, rhinitis, and gastrointestinal symptoms, whereas systemic influenza‐like symptoms such as chills, myalgia, rash, weakness, and headache are primarily associated with RSV B infection [23]. Some studies have reported that RSV A is associated with more severe clinical disease. RSV infection often leads to severe respiratory illness, resulting in hypoxemia. Compared to RSV B, RSV A infection is more likely to cause symptoms of hypoxemia [24]. Additionally, a higher proportion of patients with RSV A infection require mechanical ventilation, indicating greater disease severity [25]. Recent evidence suggests that specific genotypes of the RSV A subtype are associated with increased disease severity. The NA1 RSV A genotype has been linked to higher hospitalization rates, more severe clinical courses, and higher viral loads [26]. In contrast, BA RSV B infection tends to present with milder symptoms and is more frequently associated with eosinophilia and a family history of asthma [27]. Therefore, a deeper understanding of the distinct clinical symptoms and manifestations caused by different RSV strains will aid in the development of more effective treatment strategies and vaccine formulations to prevent infections caused by both RSV A and B subtypes.

Overall, the antigenic diversity and immune evasion capabilities of this virus make it difficult to fully prevent RSV infections and complicate the development of effective vaccines and treatments. However, a deeper understanding of the biological basis of RSV provides important insights into its interactions with the host, pathological mechanisms, and epidemiological patterns.

## 3 Epidemiology of RSV

According to the World Health Organization (WHO), there are more than 33 million cases of acute lower respiratory infections caused by RSV globally each year, leading to approximately 3.6 million hospitalizations and over 100,000 deaths, with higher morbidity and mortality rates in low‐ and middle‐income countries [28, 29]. Although RSV infection can affect people of all age groups, it is particularly severe in infants, the elderly, and immunocompromised individuals. RSV infection is most harmful to infants, especially premature infants, children with congenital heart disease, and children with chronic lung diseases. These children are at higher risk for severe lower respiratory infections, such as bronchiolitis and pneumonia, due to their underdeveloped immune systems [30]. Elderly individuals are more susceptible to RSV infection because of immune system decline, which can lead to severe respiratory diseases with higher hospitalization rates and mortality. For individuals with immune deficiencies or those receiving immunosuppressive treatment, RSV infection may result in more severe and prolonged symptoms [31].

In addition, RSV infection exhibits significant seasonal and regional variations, which are primarily influenced by climatic conditions [32]. In temperate regions, RSV infections typically peak in the winter, as cold temperatures and dry air facilitate the transmission of RSV. Moreover, colder weather leads people to spend more time indoors, where close contact increases the opportunities for the virus to spread [33]. In tropical regions, RSV tends to peak during the hottest rainy season, as increased humidity also promotes the spread of the virus [34]. Before 2020, the seasonal patterns of RSV epidemics were very consistent. However, since the onset of the COVID‐19 pandemic in early 2020, the circulation patterns of RSV and other common respiratory viruses have been disrupted. Starting in the southern United States, RSV infections began to rise in the spring of 2021 and peaked in July 2021 [35]. Due to the widespread distribution of RSV, at least 50% of infants in the United States are infected with RSV, and nearly all children are infected by the age of two [36]. In Latin America and the Caribbean, the incidence of RSV‐LRTI in children under 2 years of age is approximately 15 per 100 symptomatic cases, with ICU admission rates up to 42%, and increased mortality observed among both infants and older adults [37]. Among the elderly population in Europe, RSV infection is associated with significant hospitalization rates and mortality. However, inadequate adult surveillance systems often lead to an underestimation of the disease burden in this demographic [38]. In the Asia‐Pacific region, RSV causes significant morbidity and mortality in both pediatric and elderly, stressing the need for improved regional surveillance and vaccination strategies [39]. Furthermore, a study from Jordan found that about half of hospitalized children under 5 years were RSV‐positive, with many requiring intensive respiratory support [40]. Studies from several middle‐income countries in Latin America and Asia, including Brazil, Argentina, Chile, Mexico, and Malaysia, have shown substantial RSV‐related hospitalizations and deaths in adults aged 65 years and older [41]. Importantly, recent data from Saudi Arabia indicate that RSV is the main viral cause of LRTIs. Among infants and young children, it accounts for 40%–50% of hospitalizations [42]. Therefore, RSV, as a highly seasonal and contagious pathogen, continues to pose a persistent challenge to global public health.

## 4 Pathological Features and Clinical Manifestations of RSV

The pathophysiological process of RSV infection progresses from direct cytotoxic effects of the virus to immune‐mediated secondary tissue damage, leading to a range of clinical manifestations. These can range from mild upper respiratory symptoms to severe lower respiratory infections, such as bronchiolitis and pneumonia, which can even be life‐threatening, especially in infants, the elderly, or individuals with compromised immune function. This section primarily discusses the pathological features and clinical manifestations of RSV, providing the scientific basis for its prevention and treatment.

### 4.1 Virus Invasion and Replication

**Figure 2:**
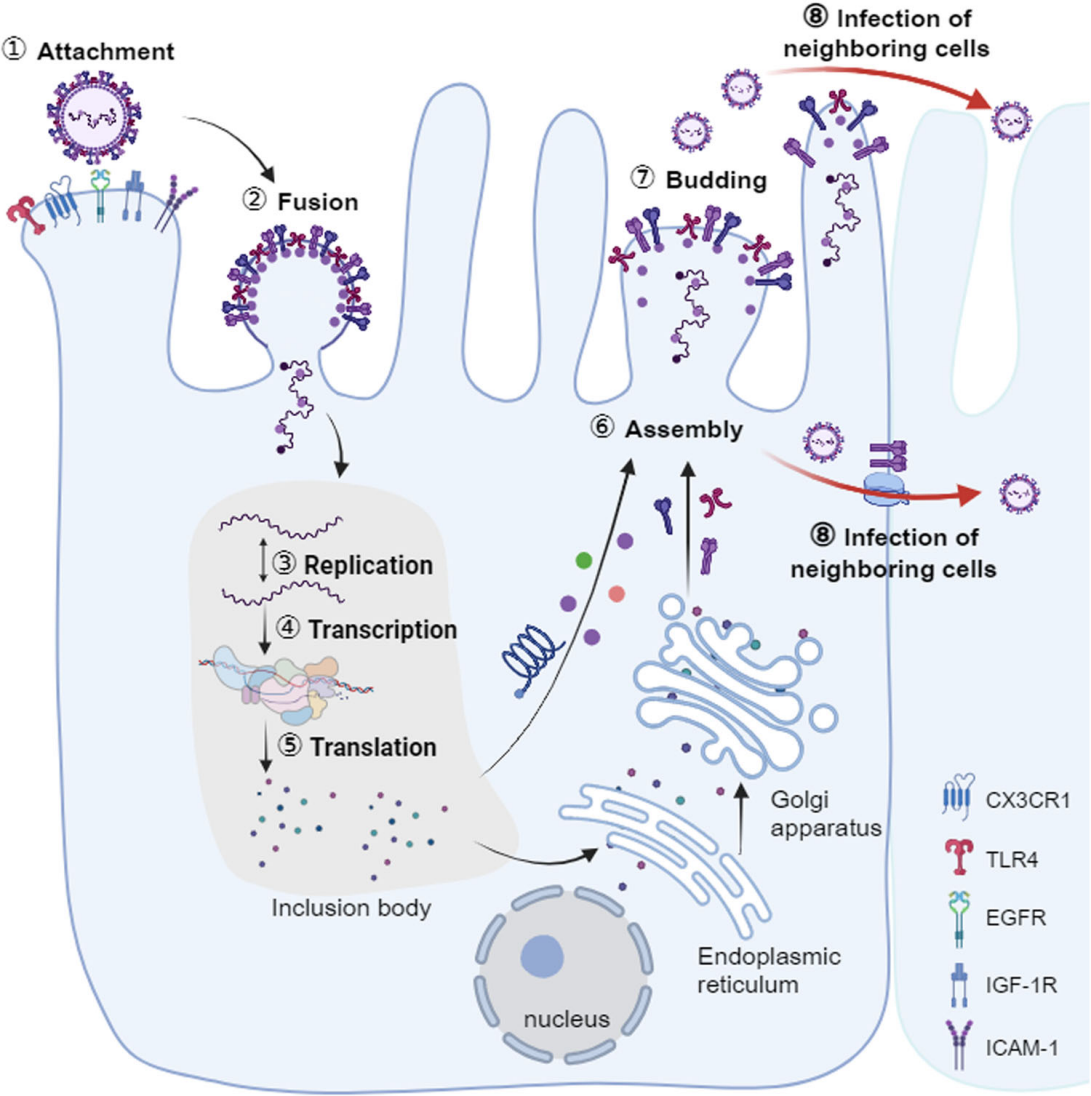
RSV invasion and spread. RSV initiates infection by binding to host cell receptors, including CX3CR1, IGF‐1R, EGFR, ICAM‐1, and TLR4. Viral entry is mediated by the F glycoprotein, which facilitates membrane fusion. Once inside the cytoplasm, RSV undergoes replication, transcription, and translation, leading to the formation of dense inclusion bodies. A portion of the translated viral proteins is directly transported, along with the viral genome, to the apical surface of the host cell for assembly, while the remaining proteins undergo further processing in the endoplasmic reticulum and Golgi apparatus. The newly assembled virions are then released either through budding or by exploiting the F protein and cytoskeletal structures to facilitate direct cell‐to‐cell transmission. [Color figure can be viewed at wileyonlinelibrary.com]

RSV enters the host through the nasal cavity or mouth, initially binding to the receptor CX3CR1 on the surface of respiratory epithelial cells via the CX3C motif of its G protein, thereby initiating the infection process [43, 44]. Subsequently, the F protein of RSV facilitates fusion and endocytosis by binding to nucleolin (NCL), epidermal growth factor receptor (EGFR), insulin‐like growth factor‐1 receptor (IGF‐1R), and intercellular adhesion molecule‐1 (ICAM‐1) [44, 45]. After the viral envelope fuses with the host cell membrane, the ribonucleoprotein (RNP) complex (viral RNA stabilized by N and P proteins) enters the host cell cytoplasm [46]. The SH protein, as a viral pore protein, promotes this process by altering membrane permeability, reducing apoptosis, and inhibiting TNF‐α signaling [47]. Once inside the host cell, the viral protein complex forms inclusion bodies in the cytoplasm, replicating the RNA genome and transcribing it into various mRNAs, which are then translated into viral proteins in the ribosomes of the host cell [48]. Newly synthesized RNA genomes and viral structural proteins assemble into new viral particles in the host cell cytoplasm. Subsequently, the viral particles exit the cell via budding, during which the virus acquires the host cell membrane, forming a new envelope [49]. The released virus can rapidly infect neighboring healthy cells, spreading throughout the respiratory tract (Figure 2).

### 4.2 Syncytium Formation and Epithelial Cell Damage

As the virus spreads in the lower respiratory tract, ciliated epithelial cells and type I alveolar cells in the respiratory tract are most susceptible to infection [50]. RSV directly damages epithelial cells during replication and release, leading to cell apoptosis and necrosis. As the infection progresses, the integrity of the epithelial cell layer is disrupted, and ciliary function is greatly diminished. The loss of cilia affects the ability of the respiratory tract to clear mucus and pathogens, making the infected area more prone to mucus accumulation, forming obstructions, and triggering clinical symptoms such as dyspnea and wheezing [51]. Furthermore, the F protein not only facilitates the viral entry into host cells but also induces membrane fusion between infected cells and neighboring cells, leading to the formation of multinucleated syncytia. By forming syncytia, RSV can spread directly to neighboring cells without leaving the host cell, avoiding exposure to the host antibodies and other immune defense mechanisms. This intercellular transmission not only exacerbates epithelial cell damage, leading to disruption of airway epithelial integrity, but also facilitates the rapid and efficient spread of the virus, increasing the severity of the infection and causing more severe LRTDs, such as bronchiolitis and pneumonia [52, 53].

### 4.3 Initiation of Inflammatory Response and Secondary Damage

The presence of the virus and the death of infected cells activate the innate immune system of the host. Epithelial cells infected with RSV recognize pathogen‐associated molecular patterns (PAMPs) of RSV, such as viral RNA, and activate downstream signaling pathways through multiple signaling cascades to secrete various pro‐inflammatory cytokines (such as IL‐6, TNF‐α) and chemokines (such as CCL5, CCL2), which stimulate the activation and recruitment of immune cells, such as neutrophils and macrophages [54]. The recruitment of these cells leads to the release of a large number of cytokines, including TNF‐α, CCL2, CCL5, IL‐6, and IL‐8, and the excessive secretion of these mediators causes secondary damage triggered by RSV infection, such as inflammation and damage to the airways and lungs, airway remodeling, airway hyperreactivity (AHR), and mucus secretion [55, 56]. TNF‐α, CCL2, and macrophages are the main factors in RSV‐induced exacerbation of asthma in mice [57]. Additionally, the recruitment of these cells to the site of infection induces an increase in the production of reactive oxygen species (ROS), leading to oxidative stress [58]. Studies have shown that oxidative stress and ROS production are key factors in causing pulmonary diseases and tissue damage associated with RSV infection in animal and cell models [59, 60]. Therefore, the excessive inflammatory response triggered by RSV infection further induces secondary tissue damage, worsening the severity of the disease.

### 4.4 Immunopathological Effects

Although immune responses help the host clear the virus, excessive immune responses can cause immunopathological damage, leading to various respiratory diseases such as bronchitis, pneumonia, and pulmonary fibrosis, and exacerbating conditions like bronchial asthma, chronic obstructive pulmonary disease (COPD), bronchiectasis, diffuse bronchiolitis, and airway remodeling [56, 61]. Infected epithelial cells release mucins, leading to increased respiratory secretions, airway obstruction, and worsening of breathing difficulties. In mouse models, RSV infection has been shown to significantly increase the activity of matrix metalloproteinase‐9 (MMP‐9) and MMP‐2 in the lungs, thereby promoting airway structural damage and pulmonary fibrosis [62]. During acute RSV infection, the sustained influx of myeloid cells into the lungs of juvenile mice can increase extracellular matrix (ECM) deposition, leading to airway wall fibrosis and promoting the development of asthma [63]. Additionally, RSV infection‐induced increases in eosinophils and lymphocytes, as well as the upregulation of TNF‐α, IFN‐γ, IL‐5, and IL‐2, are also associated with airway remodeling, which increases the risk of asthma and COPD [64].

## 5 Immune Response Induced by RSV Infection

### 5.1 Innate Immune Response

**Figure 3:**
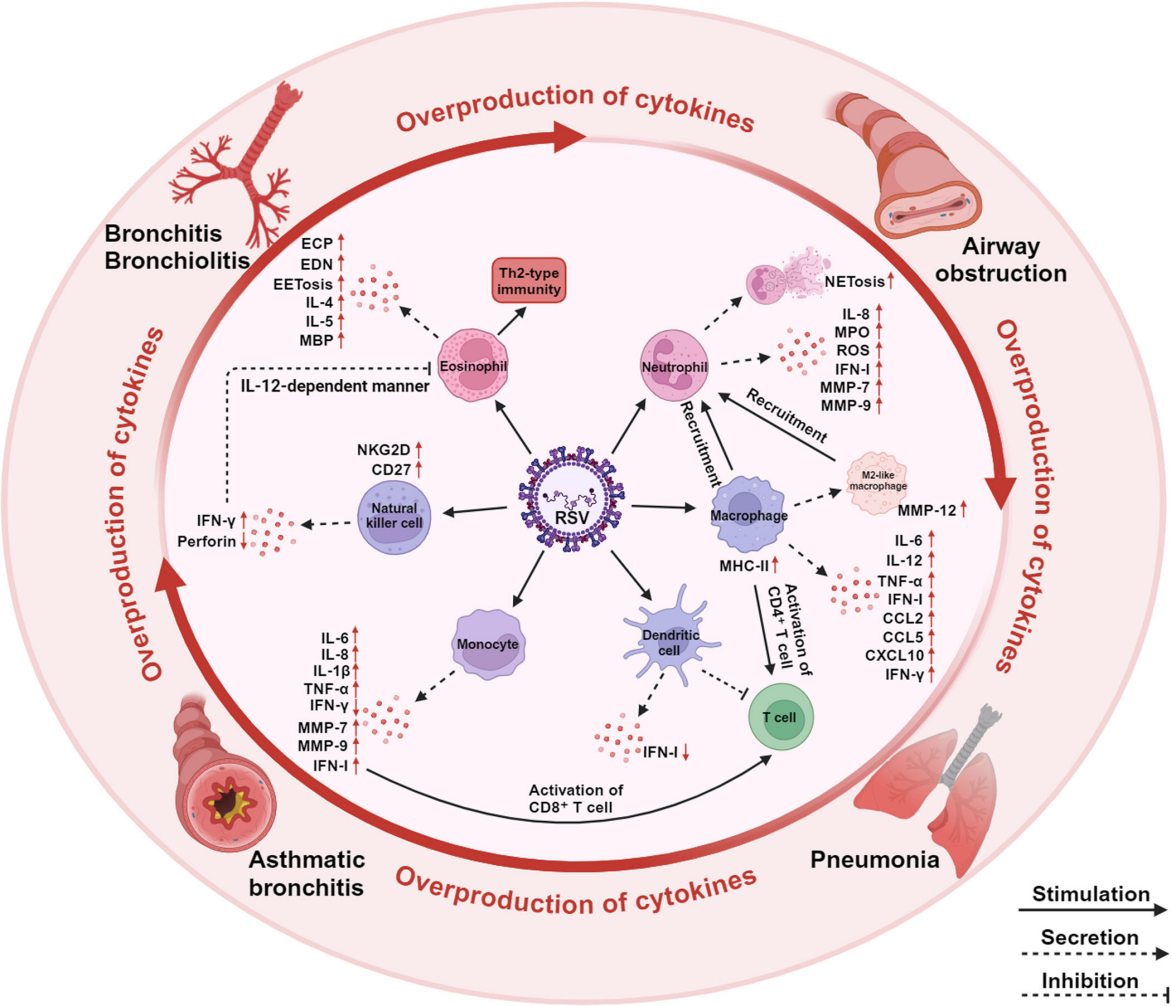
Schematic representation of the antiviral response of innate immune cells during RSV infection. Innate immune cells contribute to infection control by directly recognizing and neutralizing viral particles. However, an excessive immune response can lead to significant immunopathology and secondary tissue damage. [Color figure can be viewed at wileyonlinelibrary.com]

As respiratory epithelial cells become infected, antiviral and innate immune responses are initiated. Respiratory epithelial cells recognize RSV PAMPs, such as viral RNA, which in turn activates immune recognition pathways, including toll‐like receptors (TLRs) and RIG‐I‐like receptors. This activation induces the release of pro‐inflammatory cytokines (such as IL‐6, IL‐8, and TNF‐α), chemokines (such as CCL‐5, CCL‐3), and interferons (IFNs), which attract a large number of innate immune cells to the site of infection, initiating the antiviral immune response [65, 66, 67]. However, the excessive accumulation of these immune cells can trigger an overactive inflammatory response, leading to immunopathological damage. Innate immune cells, including neutrophils, macrophages, eosinophils, monocytes, and natural killer (NK) cells, play critical roles in defending against RSV infection (Figure 3).

#### 5.1.1 Neutrophils

Neutrophils are one of the primary defense mechanisms of the host against sterile or microbial damage. They employ three major defense mechanisms: phagocytosis, degranulation (secreting a range of antimicrobial peptides), and the formation of neutrophil extracellular traps (NETs) [68, 69]. During the early stages of RSV infection, infected respiratory epithelial cells release chemokines (such as IL‐8) to attract neutrophils to the site of infection. Once activated, neutrophils perform degranulation by releasing antimicrobial substances stored in lysosomes (such as elastase and myeloperoxidase [MPO]). These antimicrobial substances can directly attack and destroy RSV viral particles, thereby limiting their spread [70, 71]. Moreover, the RSV F protein induces NETs formation via ERK‐ and p38‐MAPK‐dependent phosphorylation pathways. Phosphorylation of ERK and p38 promotes histone citrullination and chromatin decondensation, driving NETs, which are a meshwork composed of DNA, histones, and antimicrobial proteins that trap viruses and limit their dissemination [72]. NET release is further dependent on PAD4‐mediated histone citrullination and activation of necroptosis through the receptor‐interacting protein kinase 1 (RIPK1)–RIPK3‐mixed lineage kinase domain‐like (MLKL) signaling axis. This axis not only triggers necroptosis but also amplifies NET release through MLKL phosphorylation, leading to neutrophil membrane rupture and extrusion of NET‐like structures [73, 74]. However, the literature on the role of NETs in viral diseases is contradictory [75]. Although NETs can trap viral particles, their formation has been shown to cause airway obstruction in children with severe RSV lower respiratory tract diseases (RSV‐LRTDs) [76]. In addition to degranulation and NET formation, viral particles can also be directly phagocytosed by neutrophils and destroyed through various mechanisms, such as the production of ROS [77]. However, excessive ROS release can lead to oxidative stress and lung tissue damage, exacerbating airway inflammation [58]. During RSV infection, neutrophils adhere to endothelial cells via integrins and selectins, which allows them to infiltrate lung tissue. Pulmonary neutrophilia is one of the hallmarks of RSV disease. The MyD88/TRIF signaling pathway is activated during RSV infection to recruit neutrophils to the lungs, and their activation relies on the MAVS signaling pathway‐driven inflammatory lung environment induced by IFN‐I. MAVS activates TBK1 and IKKε, which phosphorylate IRF3 and induce the production of IFN‐I. IFN‐I, in turn, upregulates the expression of neutrophil‐recruiting chemokines such as CXCL1 through a positive feedback mechanism, further promoting neutrophil infiltration [78]. Therefore, although IFN‐I has a beneficial inhibitory effect on viral load, it also drives lung inflammation and causes lung tissue damage. Thus, the potential risks of IFN‐I as a therapeutic agent for RSV infection must be carefully considered to avoid immunopathology.

#### 5.1.2 Macrophages

Macrophages, especially alveolar macrophages (AM) located on the surface of the alveolar cavity, are key innate immune cells in the respiratory tract. When RSV enters the respiratory tract, AM recognizes viral PAMPs through pattern recognition receptors (such as TLRs) on their surface and releases various pro‐inflammatory cytokines and chemokines. These factors not only contribute to direct antiviral responses but also attract other immune cells (such as neutrophils and lymphocytes) to the site of infection, further aiding in the clearance of the virus. As the infection progresses, RSV triggers AM polarization into two macrophage phenotypes, known as M1‐like and M2‐like macrophages, both of which are involved in regulating the inflammatory response. M1 macrophages promote pro‐inflammatory responses, while M2 macrophages produce anti‐inflammatory responses and repair damaged tissues during the acute and recovery phases of RSV infection [79]. Studies have shown that RSV‐infected airway epithelial cells may regulate the expression of inflammatory cytokines in AM through intercellular communication (such as the Notch‐Jagged pathway) and in cooperation with NF‐κB signaling. AM recognizes viral surface proteins via TLR4, activating the NF‐κB signaling pathway and inducing the secretion of pro‐inflammatory cytokines like IL‐6, TNF‐α, IL‐1β, and IL‐8 [80, 81]. Furthermore, AM is the primary producer of IFN‐I in the lungs during RSV infection, and IFN‐I can induce the recruitment of inflammatory monocytes to the lungs. Notably, inflammatory monocytes possess strong antiviral activity, which is crucial for controlling infection and reducing disease severity [82]. As professional antigen‐presenting cells (APCs), AM can capture and phagocytose viral particles during RSV infection. After processing the RSV antigens, macrophages present them to CD4^+^ T cells via major histocompatibility complex (MHC) class II molecules, thereby activating the adaptive immune response [83]. Besides, AM can promote the activation and proliferation of CD4^+^ T cells and their directed migration to the lung infection site by secreting inflammatory cytokines (such as IL‐12, TNF‐α, and IL‐6) and chemokines (such as CCL2, CCL5, and CXCL10) [84, 85].

Although macrophages have been shown to promote viral clearance and control immunopathology in RSV infection, the dysregulated immune response triggered by macrophages may also contribute to the development of severe disease. It is well known that IFN‐γ is a classical pathway for macrophage polarization. RSV infection may stimulate memory CD8^+^ T cells and NK cells in lung tissue to secrete excessive IFN‐γ, which induces pro‐inflammatory responses and leads to severe immunopathology [86, 87, 88]. In addition, during RSV infection, IFN‐β induces the upregulation of IL‐4Rα, which triggers M2‐like AMs to produce high levels of MMP‐12. These elevated levels of MMP‐12 accelerate the expression of CXCL1 and IL‐17A, promoting neutrophil infiltration and leading to increased AHR [89]. At the same time, in a mouse model of allergic airway disease, the levels of IFN‐γ and IL‐27 in AMs are increased, promoting the exacerbation of AHR and airway inflammation [90]. Necroptosis is considered a pro‐inflammatory form of cell death that leads to the release of intracellular contents. These contents may act as damage‐associated molecular patterns (DAMPs), exacerbating inflammation and tissue damage following viral infection [91]. Studies have shown that during RSV infection, due to viral replication and the activation of necroptosis mechanisms, macrophages produce and secrete tumor necrosis factor (TNF). TNF mediates RIPK1–RIPK3–MLKL‐dependent necroptosis of AMs in an autocrine manner, aggravating lung pathology. This pathway is triggered by TNF binding to TNFR1, forming the RIPK1–RIPK3 necrosome, which phosphorylates MLKL to cause cell lysis and DAMPs release [92]. In addition, the transforming growth factor‐β1 (TGF‐β1) is a pleiotropic cytokine that can promote inflammation depending on the environment [93]. During RSV infection, TGF‐β1 effectively suppresses the antiviral response of IFN‐I and induces mitochondrial dysfunction in AMs, promoting AM apoptosis and thereby inhibiting effective phagocytosis by AMs [94]. Therefore, it is essential to clearly understand the antiviral immune role of macrophage responses during RSV infection, which can help mitigate lung pathology and airway inflammation.

#### 5.1.3 Eosinophils

An increasing body of research evidence reveals the role of eosinophils in antiviral defense. Eosinophils are recruited to the lungs after RSV infection, where they release granule proteins with antiviral activity, such as eosinophil cationic protein (ECP) and eosinophil‐derived neurotoxin (EDN). These granule proteins have ribonuclease activity, which can degrade viral RNA, thereby inhibiting viral replication [95, 96, 97]. Eosinophils can also recognize RSV‐associated molecular patterns via a TLR7‐MyD88‐dependent pathway and coordinate the host's innate antiviral response to RSV. Mechanistically, RSV ssRNA, which serves as a ligand for TLR7, activates eosinophils, resulting in degranulation and increased expression of the phagocytic receptor CD11b. Additionally, eosinophil‐mediated accelerated RSV clearance and reduced lung dysfunction depend on the TIR adaptor molecule MyD88 and NO production by iNOS [98, 99]. Recent studies have further revealed that, during infection, eosinophils can form extracellular structures known as eosinophil extracellular traps (EETs) by releasing mitochondrial DNA and granule proteins. These structures can capture and neutralize RSV particles, limiting the spread and range of the virus [100, 101]. However, during RSV infection, the extensive infiltration of eosinophils may trigger severe inflammatory responses and lung tissue damage. RSV‐induced disease is associated with a Th2‐type immune response. Eosinophils at the site of infection promote a Th2‐type immune response by releasing cytokines such as IL‐4 and IL‐5, leading to the exacerbation of lung disease [102, 103]. In addition, while the granule proteins and peroxidases released by eosinophils have antiviral effects, they also damage the respiratory epithelial cells and tissues, resulting in lung injury and airway remodeling. The major basic protein (MBP) of eosinophil granules synergistically enhances the release of various cytokines/chemokines through interactions with airway epithelial cells, inducing bronchial hyperresponsiveness (BHR) [104, 105]. It is worth noting that in RSV vaccine research, eosinophil‐mediated adverse immune responses are also an important issue. For example, the RSV‐inactivated vaccine (FI‐RSV) from the 1960s induced abnormal Th2‐type immunity in children, accompanied by extensive eosinophil infiltration, leading to more severe pulmonary inflammation and higher mortality [106]. Therefore, eosinophils in RSV infection can both exert antiviral effects and potentially cause tissue damage due to excessive inflammatory responses and degranulation. Modulating eosinophil responses may be an important strategy to improve RSV infection outcomes and optimize vaccine design.

#### 5.1.4 Monocytes

Monocytes, especially those in the respiratory tract, are the second type of cells to infiltrate the lungs after an RSV infection. The recruitment of monocytes is crucial for controlling early RSV infection. Direct interaction of the RSV F protein with TLR4 and CD14 on the surface of monocytes further initiated innate immune pathways that resulted in the secretion of IL‐6, IL‐8, TNF‐α, and IL‐1β, which in turn mediated the antiviral immune response [107]. A recent study found that parasitic infections induce systemic mononucleosis in mice and that inflammatory monocytes protect the host from RSV infection by activating memory CD8^+^ T cells via IFN‐I signaling during microbial infection [108]. Further research revealed that during RSV infection, a lack of circulating monocytes or MyD88 deficiency is associated with reduced IFN‐I production and a decreased number of virus‐specific CD8^+^ T cells. These findings highlight the critical role of IFN‐I produced by monocytes through a MyD88‐dependent pathway in regulating CD8^+^ T cell‐mediated antiviral responses and promoting the formation of immune memory against RSV infection [109, 110]. However, another study has shown that RSV infection increased the expression of Siglec‐1 on adult monocytes, and Siglec‐1 inhibited the production of IFN‐γ by adult CD4^+^ T cells through its interaction with CD43, which is highly expressed on adult CD4^+^ T cells, thereby affecting the antiviral response [111]. Furthermore, during RSV infection, the expression of human leukocyte antigen (HLA)‐DR on monocytes is suppressed, and low expression of HLA‐DR is associated with increased disease severity [112]. Human monocytes/macrophages and myeloid dendritic cells express TLR8 and could sense RNA viruses [113]. A comparative study found that, compared to healthy infants, RSV‐infected infants had lower protein levels of TLR8 on monocytes, and low TLR8 expression may inhibit the production of early antiviral cytokines after RSV recognition, leading to more severe LRTDs in infected infants [114]. Animal model experiments have shown that juvenile mice exhibited sustained myeloid cell infiltration (including monocytes and neutrophils) after RSV infection. These cells enhanced ECM remodeling by secreting enzymes like MMPs, promoting the onset of asthma [63]. Therefore, while the recruitment of monocytes contributes to antiviral responses after RSV infection, excessive activation of monocytes may lead to secondary damage through inflammation‐mediated tissue injury and oxidative stress, thereby exacerbating the disease.

#### 5.1.5 Natural Killer (NK) Cells

Natural killer (NK) cells are key effectors of innate immunity in host defense after viral infections. They contribute to viral clearance primarily through the secretion of IFN‐γ and the elimination of infected cells via perforin‐ and granzyme‐mediated cytotoxicity, as well as antibody‐dependent cellular cytotoxicity (ADCC) [115]. Following RSV infection, NK cells accumulate in the lungs and are an important source of IFN‐γ, especially during the early stages of infection [116]. In mice infected with RSV, NK cell‐derived IFN‐γ prevents the development of pulmonary eosinophilia in an IL‐12‐dependent manner [117, 118]. However, overactivated NK cells may lead to tissue damage. During RSV infection, NK cells accumulate and become activated, expressing high levels of the activation receptors NKG2D and CD27, and producing excessive IFN‐γ, which promotes tissue immune damage [119]. High‐mobility group box‐1 (HMGB1) is an important member of the endogenous DAMPs family, which promotes persistent airway inflammation and AHR during the late stages of RSV infection [120]. Further studies have shown that RSV infection induces the formation of a complex between HMGB1 and CXCL12, which binds to CXCR4, promoting NK cell recruitment and leading to persistent airway inflammation and AHR during the later stages of RSV infection [120, 121, 122]. Animal studies have shown that IFN‐γ produced by NK cells and T cells can suppress RSV‐specific antibody responses in neonatal mice after infection, indicating that, in some cases, a strong cellular immune response may limit antibody responses during early life, suggesting that vaccines capable of inducing IFN‐γ‐secreting cells may have weaker protective effects [123]. In TLR4‐deficient mice, the recruitment of NK cells to the lungs and their cytotoxic activity were reduced after infection compared with wild‐type mice. This suggests that TLR4 plays a key role in the recruitment and functional activity of NK cells [124]. Additionally, perforin secretion is reduced in NK cells during RSV infection, which may be a mechanism by which RSV evades the host immune response [88, 125].

#### 5.1.6 Dendritic Cells (DCs)

Dendritic cells (DCs), a specialized antigen‐presenting cell, are considered a bridge between innate and adaptive immunity. During viral infection, DCs recognize the virus through innate receptors, process the viral antigens into peptides, and then present them as antigenic epitopes to T cells in complex with MHC molecules [126]. DCs are classified into conventional cDC1, cDC2, and plasmacytoid DCs (pDCs) [127]. cDC1 presents exogenous antigens to CD8^+^ T cells via MHC‐I molecules, thereby initiating cytotoxic T cell immune responses; cDC2 primarily presents antigens to CD4^+^ T cells through MHC‐II molecules, promoting the differentiation of various helper T cells such as Th1, Th2, and Th17; pDCs are responsible for the production of IFN‐I [128, 129]. IFN‐I is a major contributor to antiviral responses. pDCs recognize viral RNA through TLR‐7 and ‐8, leading to downstream activation of IRF‐7, AP1, and NF‐κB, which in turn initiates the increased production of IFN‐I [65, 130]. IFN induction in pDCs is also triggered by outside‐in signal transduction pathways via TLR7 and TLR9, as well as by the recognition of cytosolic virus‐specific patterns. TLR7 and TLR9 ligands, including single‐stranded RNA and CpG‐rich DNA, along with their synthetic derivatives, are being explored as therapeutic immune modulators that promote Th1 immune responses [131]. However, RSV infection suppresses pDCs from producing type I IFN, thereby reducing the immune response capacity of the host [131]. Additionally, during RSV infection, monocyte‐derived DCs impair T cell activation, affecting T cell proliferation and their ability to produce cytokines, thus interfering with the adaptive immune response [132].

### 5.2 Adaptive Immune Response

**Figure 4:**
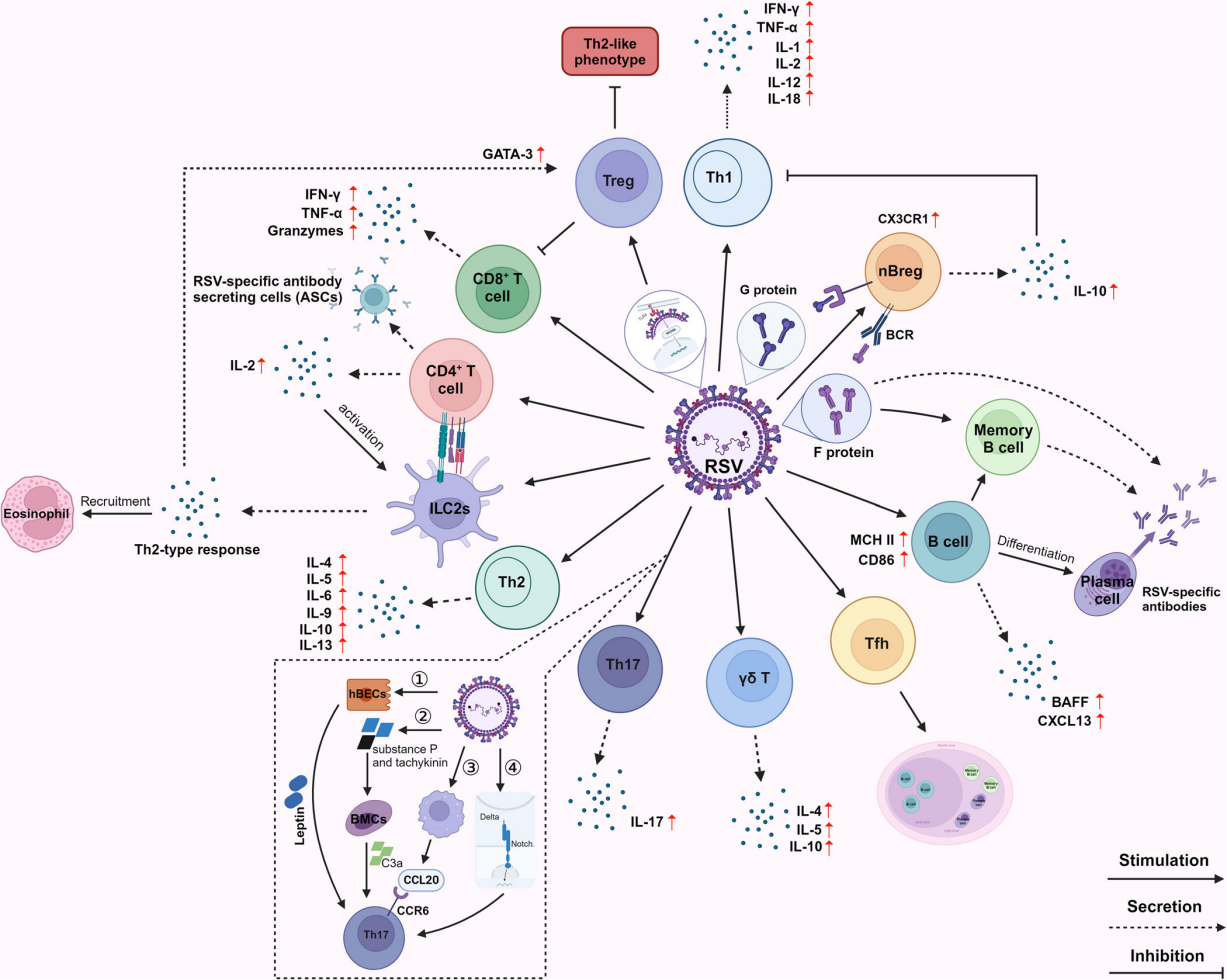
Schematic representation of the antiviral response of adaptive immune cells during RSV infection. The adaptive immune response to RSV infection primarily involves B and T lymphocytes. RSV can directly infect T and B lymphocytes, triggering the secretion of diverse cytokines that modulate the immune response. [Color figure can be viewed at wileyonlinelibrary.com]

The adaptive immune response is the second line of defense against infection. Although innate immunity responds rapidly to infection, its recognition of PAMPs is limited due to the use of germline‐encoded receptors [133]. The characteristic of adaptive immunity is that the receptors on the surface of lymphocytes are highly specific and can target pathogens to exert their effects. Activation of adaptive immune cells leads to their proliferation and clonal expansion, enabling them to perform effector functions. Ultimately, this process establishes long‐lasting immune memory in the host, which can respond rapidly upon reinfection [134, 135]. Adaptive immune response primarily involves B lymphocytes and T lymphocytes (Figure 4).

#### 5.2.1 B Lymphocytes

During RSV infection, B lymphocytes exert antiviral effects by producing antibodies. Upon infection, the innate pattern recognition receptor TLR7 recognizes RSV RNA, activates the MyD88 signaling pathway, and promotes downstream responses of NF‐κB and IRF7, leading to B‐cell proliferation, differentiation into plasma cells, and promotion of antibody (IgG and IgA) production [136]. These antibodies target the surface proteins of RSV (especially the F protein and G protein), effectively neutralizing the virus and preventing its further entry into host cells. IgA antibodies primarily induce mucosal immunity, helping to prevent the spread of the virus in the respiratory tract. Studies have shown that a deficiency in IgA may lead to recurrent RSV infections [137]. B cells also form memory B cells after infection, helping the host generate targeted antibodies more quickly upon subsequent exposure. The RSV F protein is crucial for inducing long‐term humoral and memory immune responses against RSV [138]. Studies have shown that the conformation of the RSV F protein has a direct and significant impact on the type of anti‐F protein IgG antibodies induced and the generation of F protein‐specific memory B cells, which will be important for the development of future RSV vaccines [139]. Furthermore, RSV infection upregulates the expression of B‐cell activating factor of the TNF family (BAFF) and CXCL13, which are produced through an IFN‐β‐dependent process, thereby promoting the survival and maturation of local B cells and enhancing the airway antibody response [140]. Additionally, RSV infection upregulates MHC‐II molecules and induces the expression of the costimulatory molecule CD86 on the surface of B cells [141]. CD86 enhances the interaction with T cells by binding to CD28 on the T cell surface, primarily regulating the level of T cell‐dependent antibody production by B cells and the level of cytokine production by T cells [142]. In the respiratory tract of newborns with severe RSV‐induced acute bronchiolitis, the frequency of regulatory B lymphocytes (nBreg cells) is increased. The presence of nBreg cells is associated with poor control of RSV infection and the severity of bronchiolitis in newborns [143]. The polyreactive B cell receptor of nBreg cells interacts with the RSV F protein, inducing the upregulation of the chemokine receptor CX3CR1 on the B cell surface. CX3CR1 interacts with the RSV G protein, which contains a CX3C chemokine motif, leading to the secretion of IL‐10 by nBreg cells. This, in turn, inhibits Th1 cell activation and increases the severity of lung disease [144]. Therefore, targeting nBreg cells may be a potential therapeutic strategy for treating RSV‐induced diseases.

#### 5.2.2 T Lymphocytes

##### 5.2.2.1 CD4^+^ T Lymphocytes

CD4^+^ T lymphocytes, as an important type of adaptive immune cell, play a crucial role in defending against RSV infection. CD4^+^ T cells are essential for inducing mucosal and systemic RSV‐specific antibodies and for establishing RSV‐specific IgG and IgA antibody‐secreting cells (ASCs) in the upper and lower respiratory tracts [145]. However, CD4^+^ T cells not only provide auxiliary support for antiviral immunity but may also be closely associated with diseases induced by viral infections. During RSV infection, pulmonary type 2 innate lymphoid cells (ILC2s) may act as APCs, inducing the proliferation and activation of CD4^+^ T cells via the MHC II pathway [146]. Meanwhile, activated CD4⁺ T cells secrete IL‐2, which acts on the IL‐2 receptor expressed on ILC2s, activating their JAK1/JAK3–STAT5 signaling pathway and promoting further activation of ILC2s [147]. In this activated state, ILC2s excessively produce type II cytokines such as IL‐5 and IL‐13, which in turn recruit eosinophils to the lungs, ultimately exacerbating AHR [148, 149]. However, in addition to CD4^+^ T lymphocytes, several other subtypes of cells (such as Th1, Th2, Tfh, Treg, Th17, and γδ T cells) also play distinct roles in antiviral immunity.

The Th1 subset mediates the specific adaptive immune response against intracellular pathogens and plays a key role in antiviral immunity. It induces the production of pro‐inflammatory mediators, such as IFN‐γ, IL‐1, IL‐12, IL‐2, IL‐18, and TNF‐α [150]. Among these, IL‐12 activates JAK2 and TYK2 by binding to its receptor, leading to STAT4 phosphorylation and subsequent transcriptional activation of IFN‐γ, promoting macrophage activation, enhancing the cytotoxic function of cytotoxic T lymphocytes (CTLs), and suppressing Th2‐mediated inflammatory responses [150, 151]. Studies have shown that RSV infection can activate epithelial cells to secrete IL‐23, which synergizes with IL‐12 to enhance Th1 responses. In addition, IL‐23 promotes Th17 differentiation via the IL‐23R–JAK2/TYK2–STAT3 pathway by upregulating RORγt, and drives Th2 skewing through Notch signaling‐mediated GATA‐3 upregulation, ultimately disrupting the immune balance among Th1, Th2, and Th17 cells [152, 153].

Th2 cells promote the differentiation of B cells and antibody production (particularly IgE) by secreting cytokines such as IL‐4, IL‐5, IL‐6, IL‐9, IL‐10, and IL‐13. This humoral immune response plays a crucial role in preventing viral spread. However, Th2 cells can have detrimental effects in various diseases. Th2‐dominated immune responses may lead to asthma‐like symptoms, such as increased AHR, airway mucus secretion, and eosinophil infiltration [154, 155]. Studies have shown that severe bronchiolitis induced by RSV infection is closely associated with Th2 cytokines, particularly the release of IL‐4, IL‐5, and IL‐10. Inhibition of the NF‐κB/IL‐33/ST2 pathway (such as blocking NF‐κB p65 nuclear translocation to suppress IL‐33 transcription and downstream ST2 signaling) significantly reduces the secretion of Th2 cytokines, thereby alleviating acute bronchiolitis induced by RSV infection [156]. Vaccine‐enhanced RSV disease is also associated with Th2 polarization and increased eosinophils in the lungs [157]. Th2‐biased responses, driven by GATA‐3‐mediated transcription of IL‐4, IL‐5, and IL‐13, contribute to pulmonary inflammation, eosinophilia, and increased IgG1/IgG2b antibody titers, exacerbating disease severity. In addition, T1/ST2 is a surface ligand of the IL‐1 receptor family, expressed on Th2 cells. Therefore, inhibiting T1/ST2 specifically affects the virus‐induced Th2 response, suggesting that targeting this receptor may have important clinical value for the treatment of Th2‐driven diseases [158].

Regulatory T cells (Tregs), a subset of CD4^+^ T cells, play a crucial role in regulating the pathogenesis of RSV infection [159]. RSV F protein recognizes TLR4 and increases the number of Tregs in a MyD88‐dependent or MyD88‐independent manner [107]. Previous studies have shown that during persistent viral infections, Tregs suppress CD8^+^ T cell responses [160]. In Treg‐depleted mice, the frequency of RSV‐specific CD8^+^ T cells producing IFN‐γ and TNF‐α increases, which may lead to more severe disease, including weight loss, higher morbidity, and increased airway restriction [160, 161], suggesting that Tregs can suppress excessive and pathological T cell responses. During acute RSV infection, Foxp3^+^ Tregs rapidly proliferate and accumulate in the lungs and mediastinal lymph nodes. Most pulmonary Tregs exhibit an activated phenotype (CD11a^high^, CD44^high^, CD43^glyco+^, ICOS^+^, CTLA‐4^+^) and upregulation of α4 and β7 integrin chains as well as the inhibitory molecule CTLA‐4. Tregs both promote the early recruitment of virus‐specific CD8^+^ T cells to the lung tissue and airways by modulating chemotactic signals, and suppress their activation intensity and TNF‐α production, thereby potentially alleviating RSV‐induced pulmonary immunopathology [160]. In addition, Tregs also limit Th2‐type immune responses. In Treg‐depleted mice, the expression of IL‐13 in CD4^+^ T cells and the expression of the Th2‐associated transcription factor GATA‐3 in the lungs are increased, indicating that Treg‐depleted mice exhibit a Th2‐biased response. This Th2‐biased response may be associated with AHR and more severe RSV infection [162, 163]. Tregs can also regulate B cell activity, particularly the production of anti‐F specific antibodies, which are crucial for providing protection against RSV infection. Studies have shown that Treg depletion disrupts the balance of the antibody response (anti‐F/anti‐N), impairs RSV clearance, and exacerbates immunopathogenesis [164].

Follicular helper T (Tfh) cells are a subset of CD4^+^ helper T cells involved in humoral responses [165]. Tfh cells are located in secondary lymphoid organs, including the tonsils, spleen, and lymph nodes. Their role is to trigger the differentiation of germinal center (GC) B cells into antibody‐secreting plasma cells and memory B cells [166, 167]. Therefore, Tfh cells are key to enhancing immune responses. Tfh and their promotion of high‐affinity antibody production are crucial for preventing RSV reinfection. Studies have shown that after RSV infection, Tfh‐deficient mice experience significant disease deterioration, including delayed viral clearance and increased weight loss [168]. In early life, excessive activation of the IL‐2 and STAT5 signaling pathways impairs the generation of Tfh cells, which affects the production of effective antibodies and the establishment of immune memory. Neutralization of IL‐2 helps restore immune system function, enhances Tfh cell activity, and improves antibody‐mediated immune responses [168]. Additionally, during RSV infection, activation of the ATP‐P2X7R signaling pathway may lead to the death and dysfunction of circulating Tfh (cTfh) cells, thereby impairing the ability of immune system to generate antibodies and resulting in increased disease severity [169].

Th17 cells are associated with lung inflammation and recurrent wheezing caused by RSV infection. Several mechanisms have been proposed to increase Th17 cells and IL‐17 levels. First, human bronchial epithelial cells (hBECs) infected with RSV secrete excessive leptin, promoting the differentiation of Th17 cells [170]. Second, in mice with acute RSV infection, increased release of substance P and neurokinin A leads to CD11b myeloid cells secreting C3a, which subsequently promotes an increase in IL‐17A secretion [171]. Elevated IL‐17A levels activate the Th17 pathway, exacerbating AHR [171]. Third, RSV infection damages asthma tolerance by recruiting IL‐17A‐producing cells via CCR6‐CCL20 signaling [172]. Finally, RSV activates Notch‐1/delta‐like 3 (DLL3) to stimulate Th17 differentiation in the airway microenvironment, which may be related to the onset and progression of RSV‐induced asthma [173]. Additionally, Th17 cells accumulate at mucosal sites and secrete IL‐17, which induces airway epithelial cells to produce chemotactic factors that promote leukocyte infiltration into the airways. Massive leukocyte infiltration exacerbates airway inflammation and mucus secretion [174]. Animal studies further demonstrate that IL‐17 increases primary RSV infection and airway disease by increasing mucus production, inhibiting CD8^+^ T cell activation, and reducing viral clearance [175].

During RSV infection, γδ T cells also play an indispensable role. They may promote the establishment of the immune response by producing cytokines in the early phase of respiratory infection [176]. Upon recognizing virus‐associated antigens via their TCR, γδ T cells activate the Lck‐ZAP70 signaling axis. This pathway phosphorylates the downstream adaptor proteins LAT and SLP‐76, which ultimately activate the transcription factors NFAT, AP‐1, and NF‐κB, thereby promoting the transcription and expression of the antiviral cytokines IFN‐γ and TNF‐α [177]. In the acute phase of infant RSV infection, the production of IFN‐γ by γδ T cells in peripheral blood is reduced, accompanied by an increase in Th2 cytokine production, which is associated with recurrent wheezing in patients [178]. Furthermore, studies have shown that γδ T cells produce Th1‐like cytokines early after infection, but later produce more Th2‐like cytokines such as IL‐4, IL‐5, and IL‐10. These type II cytokines exacerbate airway inflammation and lung tissue pathology during reinfection [176, 179]. Animal studies indicate that the increased severity of early RSV disease in neonatal mice is partly due to their inability to produce key inflammatory markers such as IL‐17A and IL‐1β. IL‐1β can bind to IL‐1R on γδ T cells, activating the JAK‐STAT3 signaling pathway and thereby promoting IL‐17A secretion by γδ T cells [180]. Thus, γδ T cells contribute to the early immune response through IL‐17A, representing a key component in defense against RSV infection.

##### 5.2.2.2 CD8^+^ T Lymphocytes

During RSV infection, CD8^+^ T cells play an important role in antiviral immunity. CD8⁺ T cells recognize virus‐infected cells via their TCR, activating the Lck‐ZAP70 signaling axis. This pathway phosphorylates downstream adaptor proteins LAT and SLP‐76, ultimately activating the transcription factors NFAT, AP‐1, and NF‐κB, which promote the transcription and expression of antiviral cytokines such as IFN‐γ and TNF‐α [181]. Specifically, IFN‐γ production is regulated by the transcription factor T‐bet, which is induced through the JAK‐STAT pathway under the combined signals of TCR stimulation and IL‐12, whereas TNF‐α secretion depends on downstream MAPK and NF‐κB activation mediated by TCR and costimulatory receptors such as CD28 [182]. Together, these coordinated signaling pathways enable CD8⁺ T cells to mount an effective antiviral response. Subsequently, CD8^+^ T cells kill virus‐infected cells by releasing perforin and granzymes to lyse the infected cells, or by inducing apoptosis of infected epithelial cells through the interaction of Fas with Fas ligand [183]. Studies have shown that the presence of virus‐specific CD8^+^ T cells in peripheral blood and bronchoalveolar lavage fluid (BALF) is associated with a decrease in viral titers, suggesting that virus‐specific cellular responses contribute to clearing RSV infection [184]. However, after RSV infection, the IFN‐γ and TNF produced by CD8^+^ T cells help clear the virus, but can also induce immune pathology, which results from the overproduction of IFN‐γ and TNF by memory CD8^+^ T cells in the lungs [86, 87]. Therefore, when evaluating RSV vaccine candidates, careful consideration of CD8^+^ T cell responses is necessary to balance the protective and immunopathological effects mediated by CD8^+^ T cells. Tissue‐resident memory T (T_RM_) cells have been identified as a subset of memory T cells that reside in non‐lymphoid tissues and are crucial for providing long‐term immunity [185]. CD8^+^ T cells can limit viral spread by forming long‐term immune memory. The establishment of RSV‐specific CD8^+^ T_RM_ cells in the lungs is driven by local TGF‐β and IL‐15 signaling, which upregulate integrin αE (CD103) and CD69 expression, anchoring these cells in the tissue and enhancing their survival [186]. Studies have shown that RSV‐specific CD8^+^ T_RM_ cells can provide protective immunity and help prevent secondary RSV infections [187]. Therefore, the generation of T_RM_ cells in the lungs is essential for protective immunity against RSV and should be a focus in the development of future RSV vaccines.

Overall, RSV infection triggers a complex and tightly regulated immune response involving both innate and adaptive immune cells. Innate immune cells such as neutrophils, macrophages, eosinophils, monocytes, and NK cells are essential in the early control of infection. They recognize viral particles, produce antiviral cytokines, and help clear infected cells. Subsequently, the adaptive immune response is activated, mainly through B and T lymphocytes. RSV can directly infect these cells, inducing the secretion of various cytokines that modulate the immune response and promote the production of virus‐specific antibodies. Together, these innate and adaptive mechanisms are essential for viral clearance and the resolution of infection. However, excessive or dysregulated immune activation may lead to immunopathology, including airway inflammation, tissue damage, and enhanced disease severity. These outcomes emphasize the delicate balance between protective immunity and immunopathology during RSV infection. This integrated perspective highlights the importance of understanding both the antiviral and immunomodulatory aspects of RSV‐induced immune responses, which is crucial for the development of effective vaccines and therapeutic strategies.

## 6 Previous Treatment Strategies

Evidence‐based management guidelines indicate that there is currently no effective treatment for LRTIs caused by RSV infection. Hospitalized cases still require supportive treatment, such as supplemental oxygen, respiratory support, and fluid support, but the therapeutic effects of these treatments are limited or ineffective. Fluid replacement may lead to overhydration, exacerbating pulmonary edema and respiratory distress [188]. Excessive oxygen supplementation can cause hyperoxia, which may impair alveolar macrophage function and increase the risk of ventilator‐associated pneumonia (VAP). RSV infection is more common in patients with basic conditions, such as COPD, and there is a higher likelihood of co‐infection, particularly with *Mycoplasma*, which increases the demand for noninvasive mechanical ventilation in RSV‐infected patients [189]. Due to the limitations of supportive therapy, there is a need to develop new antiviral treatment methods for RSV infection. Previously, monoclonal antibodies (mAbs) and RSV immunoglobulin were primarily used for treatment.

RSV immunoglobulin (RSV‐IGIV, RespiGam) is the first FDA‐approved immunoprophylactic agent for the prevention of severe RSV bronchiolitis in children. RSV‐IGIV is a polyclonal hyperimmune globulin with high serum RSV‐neutralizing antibody titers. RSV‐IGIV significantly reduces the RSV hospitalization rates and length of stay in premature infants and infants with bronchopulmonary dysplasia. However, RSV‐IGIV is costly, has long infusion cycles, and presents challenges, particularly in preterm infants with difficult intravenous access. Additionally, RSV‐IGIV infusion significantly increases the frequency of cyanotic episodes in patients with cyanotic congenital heart disease [190]. Therefore, the clinical use of RSV‐IGIV is limited.

Ribavirin is the only antiviral drug approved by the U.S. Food and Drug Administration (FDA) for the treatment of RSV infection. It is a broad‐spectrum guanosine analogue that inhibits the replication of both DNA and RNA viruses. Ribavirin can be administered via nebulization, intravenous injection, or oral formulation, with the nebulized form being the most commonly used. Nebulized ribavirin is typically used for the treatment of severe LRTI caused by RSV in children. The use of nebulized ribavirin has been shown to help reduce the duration of mechanical ventilation, lower the risk of mixed infections, and may be associated with a decreased incidence of recurrent wheezing [191]. However, its use is limited due to high cost, difficult administration, multiple side effects, and concerns about potential teratogenicity [192]. Additionally, the oral and intravenous forms of this drug are usually used for immunocompromised patients. Studies have shown that ribavirin treatment may have beneficial effects in reducing morbidity and mortality from RSV infections in transplant recipients or improving lung function [193, 194]. However, the bioavailability of oral ribavirin is reduced, and intravenous administration may cause acute hemolytic reactions [191]. Therefore, ribavirin has not been widely used due to its potential toxic side effects and clinical efficacy issues.

Palivizumab is the first FDA‐approved humanized mAbs for the prevention of severe RSV disease in infants and young children. It can neutralize the RSV F protein epitope, preventing the virus from fusing with epithelial cells, and its antiviral potency is 50 times greater than that of RSV‐IGIV [195]. It is active against both RSV subtypes A and B, and can be administered via intramuscular or intravenous injection, primarily for the prevention of RSV infection in high‐risk infants, including those with congenital heart disease, bronchopulmonary dysplasia, and premature birth [196, 197]. Existing studies indicate that palivizumab significantly reduces the hospitalization rate of high‐risk infants with RSV infection [198]. Additionally, palivizumab is a therapeutic option for immunocompromised patients with RSV infection, including post‐transplant patients or those with malignancies [199, 200]. However, palivizumab has high treatment costs, long treatment durations, and requires repeated injections, and hypersensitivity reactions have been observed in infants receiving the treatment [201]. Due to these characteristics, the clinical application of palivizumab is limited and restricted to children and hematopoietic stem cell transplantation (HSCT) patients [202].

Motavizumab (MEDI‐524) is a monoclonal antibody derivative of palivizumab, with higher RSV affinity and a longer half‐life [203]. Motavizumab has shown efficacy in rodent lung models of RSV infection, reducing viral titers by 50 to 100 times compared to palivizumab [204]. Studies have also found that motavizumab effectively reduces RSV hospitalization rates in high‐risk full‐term infants in the United States [205]. Despite these appealing characteristics of motavizumab, its clinical trials have been discontinued due to an increased incidence of severe side effects (allergic and hypersensitivity skin reactions), and it has not been approved by the FDA.

Due to the limitations of these antiviral therapies and preventive strategies, increasing research efforts are focused on developing new vaccines or therapies to prevent RSV infection in the population.

## 7 Future Preventive Strategies

### 7.1 Next‐Generation Monoclonal Antibodies (mAbs)

Monoclonal antibodies are often used as a preferred method to combat infections due to their high pathogen specificity [206]. In 1998, the FDA approved palivizumab, the first humanized mAb targeting the RSV F protein, for passive immunization to prevent severe LRTDs caused by RSV infection in high‐risk infants. However, its clinical application is limited and costly, as the monthly dosing requirement is restricted to preterm infants and children with congenital conditions [207]. Therefore, there is an urgent need to develop a new generation of mAbs to combat RSV infection. A new generation of RSV mAbs has been developed with an extended half‐life due to the incorporation of a YTE mutation in the Fc region of antibody, protecting all infants against LRTDs throughout the RSV season. These antibodies are more potent than palivizumab as they target epitopes in the pre‐fusion region of the RSV surface F protein [208].

A prominent example is nirsevimab (MEDI‐8897), a human mAb targeting site Ø of the RSV F protein, with a YTE mutation in the Fc region to prolong its half‐life. Moreover, nirsevimab can neutralize both RSV A and B strains, with over 50 times the potency of palivizumab [209]. Nirsevimab is administered as a single intramuscular injection during the RSV season to prevent RSV‐LRTD in infants and young children under 24 months of age [210]. In studies involving healthy late preterm and full‐term infants, nirsevimab demonstrated 75% efficacy against RSV‐LRTIs and reduced RSV‐related hospitalization rates [211]. Compared to the previously approved palivizumab, the main advantages of nirsevimab are: (1) a single intramuscular injection protects for the entire RSV season, compared to monthly dosing; (2) a single dose reduces costs; (3) it is suitable for all infants, as opposed to being limited to high‐risk infants.

Clesrovimab (MK‐1654) is a long‐acting mAb with the same YTE mutation as nirsevimab, targeting site IV of the RSV F protein. This mAb is currently in phase IIb/III clinical trials in infants. It has demonstrated approximately 50 times the potency of palivizumab in vitro, exhibits high efficacy against RSV clinical isolates, and shows equivalent activity against both RSV A and B strains. Additionally, it provides in vivo protection against RSV infection in the cotton rat prophylactic challenge model [212]. In a study evaluating the safety, tolerability, and pharmacokinetics of MK‐1654 in healthy adults, the antibody exhibited a half‐life of 73 to 88 days and an estimated bioavailability of 69% at a 300 mg dose. The overall safety profile of MK‐1654 was comparable to placebo, with a low incidence of treatment‐emergent antidrug antibodies (2.6%) and no related adverse events [213]. A model‐based meta‐analysis (MBMA) suggested a high likelihood that a single dose of MK‐1654 at ≥75 mg would achieve over 75% prophylactic efficacy for up to 5 months in infants [214]. Furthermore, a human challenge study (*n* = 70) demonstrated reduced viral load following exposure and a decrease in RSV symptomatic infection rates [214].

In general, key considerations for developing next‐generation mAbs include: (1) enhancing production efficiency or developing biosimilars to improve affordability; (2) reducing costs by lowering the required drug dose through local administration; and (3) monitoring viral resistance, which can be prevented by administering monoclonal antibody mixtures targeting multiple epitopes [215].

### 7.2 Vaccines

For viruses transmitted via the respiratory route, prior vaccination is the cornerstone of prevention. Since the 1960s, the development of an RSV vaccine has been a priority for the WHO. The year 2023 marked a significant milestone for RSV vaccines. In May, Arexvy (GSK) became the world's first approved RSV vaccine. Arexvy is a combination of the RSVPreF3 antigen (120 μg) of the RSV F protein and the AS01E adjuvant system, designed to prevent RSV‐LRTI in individuals aged 60 years and older [216]. Later that year, Abrysvo (Pfizer), the second RSV vaccine, was approved. This adjuvant‐free bivalent vaccine contains 60 μg each of RSV A and B strains and is also intended for active immunization against RSV‐LRTI in adults aged 60 and older. Additionally, Abrysvo provides passive protection to infants from birth to 6 months through maternal immunization during pregnancy [217]. In 2024, the first RSV mRNA vaccine, mRESVIA (Moderna), received approval. It uses mRNA encoding a stabilized preF protein and is designed to prevent acute lower respiratory tract infections (ALRIs) in adults aged 60 and older. However, there remains a significant unmet medical need. A search of the ClinicalTrials.gov database indicates that RSV preventive vaccine candidates are currently under clinical development through multiple strategies, including live‐attenuated vaccines, recombinant‐vector vaccines, subunit vaccines, particle‐based vaccines, chimeric vaccines, and nucleic acid vaccines [215]. There are 43 vaccines in development, of which 18 are in clinical stage development [218] (Table 1).

**Table 1:** RSV vaccine candidates under clinical development.

Candidate	Antigen	Adjuvant	Sponsor	Phase I trial	Phase II trial	Phase III trial	Results
LAV							
RSV cps2	All viral proteins	None	NIAID	2013/10/03‐2015/05; NCT01968083; NCT01852266; RSV‐seronegative children aged 6–24 months; 50 participates	—	—	Phase I: A total of 85% of vaccinees were infected with RSVcps2, 77% shed vaccine virus, 59% developed a ≥4‐fold rise in serum RSV‐neutralizing antibody titers and 68% developed a ≥4‐fold rise in anti‐RSV F IgG titers.
RSV LID/ΔM2‐2	All viral proteins except M2‐2	None	NIAID	2014/09‐2015/04; NCT02237209; NCT02040831; RSV‐seronegative children aged 6–24 months; 29 participates	—	—	Phase I: Excellent infectivity and immunogenicity, a total of 95% of vaccinees were infected with LID ΔM2‐2, 90% developed a ≥4‐fold rise in serum RSV‐neutralizing antibody titers and anti‐RSV F IgG titers.
RSV LID/ΔM2‐2/1030s	All viral proteins except M2‐2; mutation of L	None	NIAID	2016/07/15‐2017/07/07; NCT02952339; NCT02794870; RSV‐seronegative infants aged 6–24 months; 33 participates 2022/02/23‐2024/12; NCT04520659; RSV‐seronegative infants and children aged 6–24 months; 81 participates	—	—	Phase I (NCT02952339/NCT02794870): A total of 85% of vaccinees shed LID/ΔM2‐2/1030s vaccine and had ≥4‐fold rise in serum‐neutralizing antibodies; 90% of vaccinees showed an increase in anti‐RSV F IgG titers.
RSV MEDI/ΔM2‐2	All viral proteins except M2‐2	None	NIAID	2011/08‐2015/08; NCT01459198; Adults aged 18–49 years, RSV‐seropositive children aged 12–59 months, RSV‐seronegative infants and children aged 6–24 months; 60 participates	—	—	—
D46/NS2/N/ΔM2‐2‐HindIII	All viral proteins except M2‐2	None	NIAID	2017/04/06‐2018/05/25; NCT03102034; RSV‐seronegative infants and children aged 6–24 months; 32 participates	—	—	Phase I: All vaccinees were infected with vaccine; 95% shed vaccine virus. Serum RSV‐neutralizing antibodies and anti‐RSV F IgG increased ≥4‐fold in 95% and 100% of vaccines, respectively.
RSV D46cpΔM2‐2	All viral proteins except M2‐2	None	NIAID	2015/10‐2018/04/26; NCT02601612; RSV‐seropositive children aged 12–59 months and RSV‐seronegative infants and children aged 6–24 months; 30 participates	—	—	—
RSVt	All viral proteins except M2‐2	None	Sanofi	—	2023/02/06‐2025/05/21; NCT05687279; Infants and toddlers aged 6–23 months; 80 participates	2024/02/06‐2027/12/07; NCT06252285; Infants and toddlers aged 6–21 months; 6300 participates 2024/11/25‐2026/04/17; NCT06705140; Infants and toddlers aged 6–21 months; 947 participates	—
RSV 276	All viral proteins except M2‐2	None	NIAID	2017/09/22‐2020/10/01; NCT03227029; NCT03422237 RSV‐seronegative children aged 6–24 months; 65 participates 2019/05/16‐2024/04/25; NCT03916185; RSV‐seronegative children aged 6–24 months; 67 participates	—	—	Phase I (NCT03227029/NCT03422237): A total of 96% of vaccinees were infected with RSV/276, 92% developed a ≥4‐fold rise in serum RSV‐neutralizing antibody titers and anti‐RSV F IgG titers.
RSV 6120/∆NS1	All viral proteins except NS1	None	NIAID	2018/06/25‐2024/09/30; NCT03596801; RSV‐seropositive children aged 12–59 months and RSV‐seronegative infants and children aged 6–24 months; 60 participates	—	—	—
RSV 6120/F1/G2/∆NS1	All viral proteins except NS1; modification of F	None	NIAID	2018/06/25‐2024/09/30; NCT03596801; RSV‐seropositive children aged 12–59 months and RSV‐seronegative infants and children aged 6–24 months; 60 participates	—	—	—
RSV ΔNS2/Δ1313/I1314L	All viral proteins except NS2 and codon in L; modification at codon 1314	None	NIAID	2017/09/22‐2020/10/01; NCT03227029; NCT03422237; RSV‐seronegative children aged 6–24 months; 65 participates 2013/06/01‐2025/10/01; NCT01893554; RSV‐seropositive children aged 12–59 months, RSV‐seronegative infants and children aged 6–24 months, and infants aged 4–6 months; 88 participates 2019/05/16‐2024/04/25; NCT03916185; RSV‐seronegative children aged 6–24 months; 67 participates	2020/09/17‐2023/04/13; NCT04491877; Children aged 6–18 months; 259 participates	—	Phase I (NCT03227029/NCT03422237): A total of 88% of vaccinees were infected with RSV/ΔNS2/Δ1313/I1314L, 60% developed a ≥4‐fold rise in serum RSV‐neutralizing antibody titers and anti‐RSV F IgG titers. Phase I (NCT01893554): This vaccine was well tolerated, infectious (RSV/ΔNS2/Δ1313/I1314L replication detected in 90% of vaccinees), and immunogenic (geometric mean serum RSV plaque‐reduction neutralizing antibody titer, 1:64). Phase II: This vaccine boosted serum RSV A‐neutralizing antibody titers and anti‐RSV F IgG antibody titers.
RSV 6120/ΔNS2/1030s	All viral proteins except NS2; modification of L	None	NIAID	2017/10/13‐2021/05/31; NCT03387137; RSV‐seropositive children aged 12–59 months and RSV‐seronegative infants and children aged 6–24 months; 45 participates 2019/05/16‐2024/04/25; NCT03916185; RSV‐seronegative children aged 6–24 months; 67 participates	—	—	Phase I (NCT03387137): RSV/6120/ΔNS2/1030s infected 100% of RSV‐seronegative vaccinees and was immunogenic and genetically stable.
CodaVax‐RSV	All viral proteins; modifications of the genome	None	Codagenix	2020/07/10‐2021/05/26; NCT04295070; Adults aged 50–75 years; 36 participates 2023/03/28‐2025/08/27; NCT04919109; RSV‐seropositive children aged 2–5 years and RSV‐seronegative children aged 6 months to <2 years; 51 participates	—	—	—
MV‐012‐968	All viral proteins except G and SH; codon deoptimization NS1 and NS2	None	Meissa Vaccines	2021/06/03‐2023/10; NCT04909021; RSV‐seronegative children aged 6–36 months; 63 participates 2020/01/14‐2020/08/27; NCT04227210; Adults aged 18–40 years; 20 participates 2020/06/09‐2021/05/07; NCT04444284; RSV‐seropositive children aged 15–59 months; 34 participates	2020/12/29‐2021/09/09; NCT04690335; Adults aged 18–45 years in the human viral challenge model; 60 participates	—	Phase I (NCT04909021): In the highest dose group, 78% of children developed a serum neutralizing antibody response, and 89% of children exhibited an IgA response in the nasal mucosa.
VAD00001	All viral proteins; attenuation method	None	Sanofi	2020/09/17‐2023/04/13; NCT04491877; Children aged 6–18 months; 259 participates	—	—	—
Recombinant‐vector vaccines							
ChAd155‐RSV (GSK3389245A)	F protein; N protein; M2‐1 protein	None	GSK	2015/07/23‐2017/01/26; NCT02491463; Adults aged 18–45 years; 73 participates	2017/01/11‐2020/11/26; NCT02927873; RSV‐seropositive infants aged 12–23 months; 107 participates 2019/04/08‐2021/07/22; NCT03636906; Infants aged 6–7 months; 201 participates	—	Phase I: ChAd155‐RSV generated increases in specific humoral and cellular immune responses without raising significant safety concerns, including RSV‐A neutralizing antibodies, anti‐F IgG titers, the frequency of circulating B‐cells secreting anti‐F IgG/IgA and RSV‐F‐specific IFN‐γ‐secreting T‐cells. Phase II (NCT02927873): The vaccine boosted RSV F‐specific IgG and RSV‐A neutralizing antibody levels across low, medium, and high concentrations. Phase II (NCT03636906): ChAd155‐RSV administered to 6‐ to 7‐month olds had a reactogenicity and safety profile, showed no evidence of VAERD, and induced a humoral immune response.
BLB‐201	F protein	None	Blue Lake Biotechnology	2022/07/20‐2023/05/03; NCT05281263; Adults aged 18–75 years; 30 participates	2023/03/09‐2024/12/23; NCT05655182; RSV seronegative and seropositive infants and children aged 6 months to 5 years; 137 participates	—	Phase I: Nasal RSV‐specific IgA responses were detected in 48%; RSV‐neutralizing antibodies titers in serum rose ≥1.5‐fold. Peripheral blood RSV F‐specific CD4^+^ and CD8^+^ T cells increased from ≤0.06% at baseline to ≥0.26% and 0.4%, respectively, in >93% participants.
MVA‐BN‐RSV	F protein; G protein; N protein; M2 protein	None	Bavarian Nordic	2015/08‐2016/05; NCT02419391; Adults aged 18–65 years; 63 participates	2016/09‐2018/12; NCT02873286; Adults aged ≥55 years; 420 participates 2021/02/22‐2021/11/02; NCT04752644; Adults aged 18–50 years; 73 participates	—	Phase I: MVA‐BN‐RSV induced robust T cell responses covering all 5 inserts with fold increases ranging from 1.8 to 3.8. Higher and broader responses were observed in the high dose groups. Moderate but consistent humoral responses were observed against A and B RSV subtypes. Phase II (NCT02873286): A single dose increased the levels of neutralizing (RSV A and B) and IgG/IgA antibodies and induced a broad Th1‐biased cellular immune response (IFN‐γ ELISPOT) to all 5 vaccine inserts. Antibody responses remained above baseline for 6 months. A 12‐month booster dose elicited a booster effect in antibody and T‐cell responses. Phase II (NCT04752644): Vaccine efficacy against symptomatic, laboratory‐confirmed or culture‐confirmed infection was 79.3% to 88.5%. Serum IgA and IgG titers increased approximately 4‐fold after MVA‐BN‐RSV vaccination. IFN‐γ‐producing cells increased 4‐ to 6‐fold after MVA‐BN‐RSV in response to stimulation with the encoded RSV internal antigens.
Ad26.RSV.preF	F protein	None	Janssen Vaccines and Prevention B.V.	2017/11/29‐2020/04/21; NCT03303625; Adults aged 18–50 years and RSV‐seropositive toddlers aged 12–24 months; 48 participates 2019/01/21‐2021/11/02; NCT03606512; RSV‐seronegative toddlers aged 12–24 months; 38 participates 2016/11/08‐2019/01/29; NCT02926430; Adults aged ≥60 years; 73 participates 2019/01/07‐2019/07/28; NCT03795441; Adults aged ≥18 years; 24 participates	2017/10/16‐2018/11/27; NCT03334695; Adults aged 18–50 years; 64 participates	—	Phase I (NCT03303625): GMT for RSV A2 neutralization increased from 121 to 1608 at day 29, and 2235 at day 57, remaining elevated over 7 months. Respiratory syncytial virus infection was confirmed in fewer children receiving Ad26.RSV.preF than placebo. Phase I (NCT03606512): Administering three doses of vaccine on days 1, 29, and 57 boosted RSV preF/postF‐specific IgG, RSV‐A2 neutralizing antibody levels on days 85 and 267. Phase I (NCT02926430): Following the initial vaccination, GMTs for RSV‐A2 neutralizing antibodies increased significantly from baseline levels (432 for LD and 512 for HD) to day 29 (1031 for LD and 1617 for HD). F‐specific IFN‐γ‐secreting T cells also increased substantially from baseline. These immune responses were still maintained above baseline levels 2 years after immunization and could be boosted with a second immunization at 1 year. Phase II: Post‐challenge, viral load, RSV infections, and disease severity were lower in Ad26.RSV.preF recipients. From baseline to 28 days postimmunization, geometric mean fold increases in RSV A2 neutralizing antibody titers of 5.8 were observed in Ad26.RSV.preF.Ad26.RSV.preF was well tolerated.
VXA‐RSV‐f	F protein	dsRNA	Vaxart	2016/06/22‐2017/09/20; NCT02830932; Adult aged 18–49 years; 66 participates	—	—	—
**Subunit vaccines**							
RSVPreF3 OA	F protein	AS01E	GSK		2020/12/09‐2021/10/25; NCT04657198; Adults aged ≥60 years; 126 participates 2024/09/19‐2026/03/31; NCT06597916; Immunocompromised patients aged ≥18 years; 200 participates 2023/07/28‐2025/06/05; NCT05921903; Adults aged ≥50 years and immunocompromised patients aged ≥18 years (received lung and spleen transplant); 387 participates	2021/05/25‐2024/05/31; NCT04886596; Adults aged ≥60 years; 26668 participates 2022/10/28‐2024/04/03; NCT05590403; Adults aged ≥60 years and adults aged 50–59 years at increased risk of RSV‐LRTD; 1544 participates 2024/08/05‐2025/09/12; NCT06551181; Adults aged ≥60 years; 2600 participates 2024/08/01‐2026/09/30; NCT06534892; Adults aged ≥60 years; 12000 participates 2024/10/01‐2025/10/28; NCT06614725; Adults aged ≥60 years and adults aged 50–59 years at increased risk of RSV‐LRTD; 750 participates 2024/04/29‐2025/04/28; NCT06389487; Adults aged ≥ 60 years and adults aged 18–49 years at increased risk of RSV‐LRTD; 1457 participates	Phase II (NCT04657198): The vaccine high dose adjuvanted with AS01E boosted RSVPreF3‐specific IgG antibody, RSV‐A/B neutralizing antibody, and frequencies of RSVPreF3‐specific CD4^+^ T cells. Phase III (NCT04886596): Efficacy over 2 seasons of one RSVPreF3 OA dose was 67.2% against RSV‐LRTD and 78.8% against severe RSV‐LRTD. Efficacy over 2 seasons of a first dose followed by revaccination was 67.1% against RSV‐LRTD and 78.8% against severe RSV‐LRTD. Phase III (NCT05590403): In adults HA‐RSV group (adults aged 50–59 years) and adults AIR‐RSV group (adults aged 50–59 years at increased risk of RSV‐LRTD), the vaccine showed higher neutralizing antibody titers against RSV‐A and RSV‐B, as well as an increased frequency of RSVPreF3‐specific CD4^+^ and CD8^+^ T cells, one month post‐administration compared to the OA‐RSV group (adults aged ≥60 years).
RSVpreF	F protein	None	Pfizer	2023/06/22‐2024/02/29; NCT05900154; Children aged 2–17 years; 128 participates	2019/08/07‐2021/09/30; NCT04032093; Pregnant women aged 18–49 years; 1153 participates 2020/11/10‐2021/08/16; NCT04785612; Adults aged 18–50 years; 70 participates	2021/10/21‐2022/04/04; NCT05096208; Adults aged 18–49 years; 1028 participates 2020/06/17‐2023/10/27; NCT04424316; Infants born to women vaccinated during pregnancy aged 18–49 years; 14699 participates 2023/05/11‐2024/03/18; NCT05842967; Adults aged ≥18 years at high risk of severe RSV disease; 886 participates 2024/10/07‐2025/03/01; NCT06593587; Adults aged ≥60 years; 360 participates 2021/08/31‐2026/06/12; NCT05035212; Adults aged ≥60 years; 45000 participates 2024/03/12‐2025/07/24; NCT06325657; Pregnant participants with HIV and their infants (aged 0–49 years); 343 participates	Phase II (NCT04032093): RSVpreF vaccine elicited neutralizing antibody responses with efficient transplacental transfer and without evident safety concerns. Phase II (NCT04785612): After participants were inoculated with the challenge virus, vaccine efficacy of 86.7% was observed for symptomatic RSV infection confirmed by any detectable viral RNA on at least 2 consecutive days. The median AUC for the RSV viral load as measured by RT‐qPCR assay was 0.0 in the vaccine group and 96.7 in the placebo group. The geometric mean factor increases from baseline in RSV A‐neutralizing titers 28 days after injection was 20.5 in the vaccine group and 1.1 in the placebo group. This vaccine was effective against symptomatic RSV infection and viral shedding. No evident safety concerns were identified. Phase III (NCT05096208): This vaccine boosted neutralizing antibody titers against RSV‐A and RSV‐B. Phase III (NCT04424316): This vaccine attenuated percentage of severe medically attended lower respiratory tract illness (MA‐LRTI) cases due to RSV in infants from birth to 180 days of age.
BARS13	G protein	AE011	Advaccine Biopharmaceuticals	2018/10/16‐2019/08/02; NCT04851977; Adults aged 18–45 years; 60 participates	2021/05/24‐2024/04/02; NCT04681833 Adults aged 60–80 years; 125 participates	—	Phase I: Generally good safety and tolerability profile, no significant difference in terms of adverse reaction severity or frequency was observed between different dose groups. Phase II: Levels of anti‐G antibodies exhibited a dose‐ and frequency‐dependent responses in the older population.
RSVPreF3 (GSK3844766A)	F protein	AS01 (AS01E or AS01B)	GSK	2019/09/25‐2020/12/11; NCT04090658; Japanese adults aged 60–80 years; 40 participates	2019/01/21‐2020/11/30; NCT03814590; Adults aged 18–80 years; 1053 participates	—	Phase I: The vaccine boosted RSVPreF3‐specific IgG antibody and RSV‐A neutralizing antibody titers at days 31, 61, 91. Phase II: The vaccine boosted RSVPreF3‐specific IgG antibody and RSV‐A neutralizing antibody, which increased in an antigen concentration‐dependent manner and were highest after dose one. Compared to pre‐vaccination, the geometric mean frequencies of polyfunctional CD4^+^ T cells increased after each dose and were significantly higher in adjuvanted than unadjuvanted vaccinees.
SCB‐1019	F protein	None	Clover Biopharmaceuticals	2023/12/13‐2025/05; NCT06194318; Adults aged 18–85 years; 60 participates	—	—	Phase I: Preliminary results indicated that SCB‐1019 exhibited good safety and immunogenicity. On day 28 post‐immunization, the adjuvant‐free SCB‐1019 induced GMTs of neutralizing antibodies against RSV‐A and RSV‐B of approximately 30,500 IU/mL and 32,000 IU/mL, respectively.
DPX‐RSV(A)	SH protein	DepoVax	Dalhousie University	2015/05‐2017/03/14; NCT02472548; Adults aged 50–64 years; 40 participates	—	—	Phase I: Robust anti‐SHe‐specific immune responses were demonstrated in the DPX‐RSV(A) 10 and 25 μg groups, and responses were sustained in the DPX‐RSV(A) 25‐μg group at day 421. This vaccine was highly immunogenic, with sustained antigen‐specific antibody responses, and had an acceptable safety profile.
RSV‐F	F protein	None	Novavax	—	2014/10‐2016/03; NCT02266628; Adults aged ≥60 years; 1599 participates	2015/11‐2016/12; NCT02608502; Older adults aged ≥60 years; 11850 participates	—
MKK900	F protein	MA103	Maxvax Biotechnology	2023/11/13‐2027/02/28; NCT06642558; Adults aged ≥18 years; 522 participates	—	—	—
LYB005	F protein	None	Guangzhou Patronus Biotechnology	2024/07‐2025/04; NCT06442241; Adults aged ≥18 years; 84 participates	—	—	—
MEDI7510	F protein	GLA‐SE	MedImmune	2014/04‐2015/06; NCT02115815; Adults aged 60–99 years; 246 participates 2015/01/05‐2016/02/24; NCT02289820; Adults aged 60–99 years; 363 participates	—	—	Phase I (NCT02115815): At the highest dosage level with adjuvant, half of the subjects had a ≥3‐fold rise from day 0 in RSV neutralizing antibody titers, and all had a ≥3‐fold rise in antibody levels in anti‐F IgG on day 29. 74% of subjects in the highest dosing cohort had a ≥3‐fold rise in F protein‐specific IFN‐γ‐producing T cells. Phase I (NCT02289820): High‐dose group vaccine boosted RSV F‐specific IgG, RSV‐A neutralizing antibody levels, and the number of F protein‐specific IFN‐γ‐producing T cells.
DS‐Cav1	F protein	Alum	NIAID	2017/02/22‐2019/10/03; NCT03049488; Adults aged 18–50 years; 95 participates	—	—	Phase I: DS‐Cav1 vaccination elicited a robust boost in RSV F‐specific antibodies and neutralizing activity that was sustained above baseline for at least 44 weeks.
VN‐0200	VAGA‐9001a	MABH‐9002b	Daiichi Sankyo	—	2022/10/13‐2024/02/15; NCT05547087; Japanese adults aged 60–80 years; 342 participates	—	—
RSV Maternal vaccine (GSK3888550A)	F protein	None	GSK	2018/10/30‐2019/09/02; NCT03674177; Nonpregnant women aged 18–45 years; 502 participates	2019/11/05‐2021/05/14; NCT04126213; Pregnant women aged 18–40 years; 534 participates	—	Phase I: The vaccine boosted RSVPreF3‐specific IgG antibody and RSV‐A neutralizing antibody, with medium to high doses demonstrating substantially greater immunogenicity compared to low doses. Phase II: The vaccine enhanced the levels of RSV MAT‐specific IgG antibodies, RSV‐A neutralizing antibodies, and RSV‐B neutralizing antibodies in maternal subjects and their infants, and these antibodies were sustained for at least 6 months.
**Particle‐based vaccines**							
RSV F nanoparticle vaccine	F protein	Aluminum	Novavax	2014/11‐2016/04; NCT02296463; Children aged 24–72 months; 32 participates	2012/10‐2013/05; NCT01704365; Adults aged 18–35 years; 330 participates 2014/09‐2016/07; NCT02247726; Third‐trimester pregnant women aged 18–40 years; 50 participates 2013/10‐2014/04; NCT01960686; Women aged 18–35 years; 720 participates 2015/10‐2016/11; NCT02593071; Adults aged ≥60 years; 1330 participates	2015/12‐2019/07; NCT02624947; Third‐trimester pregnant women aged 18–40 years; 4636 participates	Phase II (NCT01704365): Anti‐F IgG antibodies rose 6.5–15.6‐fold, with significantly higher levels in 2‐dose, adjuvanted regimens at day 56. A 2.7‐ and 3.5‐fold rise in RSV/A and RSV/B microneutralization antibodies were noted at day 56. Phase II (NCT02247726): RSV‐specific antibody levels increased significantly among vaccine recipients, including responses competitive with well‐described monoclonal antibodies specific for multiple RSV neutralizing epitopes. Transplacental antibody transfer was 90%–120% across assays for infants of vaccinated women. Women with an interval of ≥30 days between vaccination and delivery demonstrated higher placental antibody transfer rates than women with an interval <30 days. Half‐lives of RSV‐specific antibodies in infants approximated 40 days. Phase II (NCT01960686): RSV F nanoparticle vaccine formulations were well tolerated and immunogenic. The optimal combination of convenience and rapid response for immunization in the third trimester occurred with 120 μg RSV F and 0.4 mg aluminum, which achieved peak immune responses in 14 days and sufficient persistence through 91 days to allow for passive transfer of IgG antibodies to the fetus. Phase III: Maternal vaccination with RSV F‐nanoparticle vaccine was safe and immunogenic; maternal immunization was associated with reduced risk of RSV‐confirmed MS‐LRTI and LRTI with severe hypoxemia in early infancy.
SynGEM	F protein	None	Mucosis B.V.	2016/10‐2017/12; NCT02958540; Adults aged 18–49 years; 48 participates	—	—	Phase I: SynGEM induced systemic plasmablast responses and significant, durable increases in RSV‐specific serum antibody in healthy, seropositive adults.
V‐306	F protein	Pam2Cys	Virometix	2020/09/07‐2022/03/02; NCT04519073; Adult women aged 18–45 years; 60 participates	—	—	Phase I: In the medium‐ and high‐dose groups, V‐306 induced an increase in FsIIm‐specific IgG titers, which lasted at least 4 months.
IVX‐121	F protein	Aluminum salts	Icosavax	2021/07/19‐2022/03/12; 2020‐003633‐38; Adults aged 18–75 years; 220 participates	—	—	Phase I: Within 12 months after a single administration of IVX‐121, the neutralizing antibody response against RSV is highly persistent. One month after revaccination, IVX‐121 induced a strong RSV‐A immune response with no increase in RSV‐B titers.
**Chimeric vaccines**							
rBCG‐N‐hRSV	N protein	None	Pontificia Universidad Catolica de Chile	2017/06/27‐2018/06/01; NCT03213405; Adult males aged 18–50 years; 24 participates	—	—	Phase I: The rBCG‐N‐RSV vaccine was safe, well‐tolerated, and no serious adverse events related to the vaccine were recorded. Serum IgG‐antibodies directed against *Mycobacterium* and the N‐protein of RSV increased after vaccination, which were capable of neutralizing RSV in vitro. Additionally, all volunteers displayed increased cellular response consisting of IFN‐γ and IL‐2 production against PPD and the N‐protein, starting at day 14 and 30 postvaccination respectively.
SeV/RSV	F protein	None	NIAID	2018/05/16‐2019/02/14; NCT03473002; Adult aged 18–45 years; 21 participates	—	—	Phase I: The geometric mean fold rise (GMFR) for SeV‐specific antibody was 2.0 and for RSV F‐specific antibody was 1.1 in the vaccine group. The vaccine was well‐tolerated. No severe reactions occurred.
**Nucleic acid vaccines**							
IN006	F protein	None	Innorna	2024/11/11‐2027/04; NCT06645665; Adults aged ≥18 years; 240 participates 2025/08‐2026/10; NCT06287450; Adult aged 18–79 years; 200 participates	—	—	—
STR‐V003	F protein	None	Starna Therapeutics	2024/05‐2025/05; NCT06344975; Adults aged ≥18 years; 48 participates	—	—	—
mRNA‐1345	F protein	None	Moderna	2020/09/30‐2024/07/18; NCT04528719; Adults aged 18–79 years and children aged 12–59 months; 651 participates	2023/10/24‐2025/06/06; NCT06097299; Children aged 2–17 years at high‐risk of RSV disease; 340 participates 2023/11/15‐2026/02/18; NCT06143046; Pregnant women aged 18–40 years; 360 participates	2021/11/17‐2025/08/25; NCT05127434; Adults aged ≥60 years; 36814 participates 2023/10/06‐2026/07/30; NCT06067230; High‐risk adults aged ≥18 to <60 years; 1150 participates	Phase I (NCT04528719): At one month postinjection, a single injection of mRNA‐1345 boosted RSV‐A and RSV‐B neutralizing antibody titers and RSV preF‐binding antibody concentrations. Antibody levels remained above baseline through 12 months. A 12‐month booster injection also increased RSV‐A and RSV‐B neutralizing antibody titers and preF‐binding antibody concentrations. Phase III (NCT05127434): Vaccine efficacy was 83.7% against RSV‐associated lower respiratory tract disease with at least two signs or symptoms and 82.4% against the disease with at least three signs or symptoms. Vaccine efficacy was 68.4% against RSV‐associated acute respiratory disease. mRNA‐1345 enhanced RSV‐A and RSV‐B neutralizing antibody titers and RSV preF‐binding antibody concentrations in adults (≥60 years) across various subgroups, including those at risk for severe disease.
JCXH‐108	Unclear	Unclear	Immorna Biotherapeutics	2024/09/25‐2025/03; NCT06564194; Adults aged ≥18 years; 75 participates	—	—	—

#### 7.2.1 Live‐Attenuated Vaccines

Live‐attenuated vaccines (LAVs) mimic natural infection to trigger a strong immune response, including mucosal antibodies and cellular immunity while being attenuated to reduce virulence. The advantage of LAV for RSV in infants and young children is that they can replicate in the respiratory tract and elicit robust humoral and cellular responses, even in the presence of maternally acquired antibodies [219]. However, LAV may be limited to the pediatric population under 2 years of age, as pre‐existing immunity in older individuals may prevent sufficient replication to generate a protective immune response. Additionally, the challenge for LAV lies in achieving attenuation while maintaining sufficient immunogenicity to elicit a strong immune response [218]. Three key modifications to the RSV genome have been achieved using reverse genetics technology: the ∆M2‐2 deletion, which attenuates viral replication and enhances viral gene transcription and antigen expression [220], and the ΔNS1/ΔNS2 deletion, which boosts IFN‐I signaling and antiviral gene expression [221, 222]. LAV candidates attenuated by M2‐2, NS1, or NS2 deletion exhibit excellent immunogenicity, inducing elevated serum RSV‐neutralizing antibodies in vaccine recipients.

#### 7.2.2 Recombinant‐Vector Vaccines

Recombinant vector vaccines employ a modified replication‐defective virus to deliver RSV antigen genes, thereby stimulating humoral and cellular immunity [215]. Five candidates are currently in clinical trials for children and elderly populations. The first candidate, MVA‐BN‐RSV, is a novel poxvirus‐vectored vaccine encoding RSV surface proteins F and G (A and B subtypes) and internal proteins N and M2 within the MVA‐BN vector [223]. In a Phase I clinical trial, no differences were observed in safety or immunogenicity between the adult and elderly groups. The well‐tolerated MVA‐BN‐RSV candidate vaccine induced broad cellular and humoral immune responses [223]. In a Phase IIa human challenge trial, MVA‐BN‐RSV vaccination resulted in reduced viral load and fewer confirmed infections [224]. A Phase II clinical trial in elderly individuals demonstrated that MVA‐BN‐RSV induced a broad immune response lasting at least 6 months, with the potential to be boosted at 12 months [225]. Moreover, in a Phase III clinical study, MVA‐BN‐RSV demonstrated moderate protection against RSV‐associated LRTDs in elderly individuals [226]. Second, BLB‐201 is a live viral vector intranasal RSV candidate vaccine based on parainfluenza virus 5 (PIV5) encoding the RSV F antigen. A phase I clinical study demonstrated that BLB‐201 had a favorable safety and immunogenicity profile in RSV‐seropositive adults, supporting its continued clinical development [227]. Third, VXA‐RSV‐f is an adenoviral vector‐based vaccine candidate. Phase I clinical trials of this vaccine (NCT02830932) have been completed and results are not yet available.

The four vector‐based candidate, Ad26.RSV.preF is an adenovirus serotype 26 (Ad26) vector encoding the RSV F protein stabilized in its preF conformation, designed for use in older adults and children. In a Phase I/IIa study, Ad26.RSV.preF demonstrated good tolerability and robust immunogenicity in RSV‐seronegative infants and induced durable humoral and cellular immune responses following a single immunization in older adults [228, 229]. The vaccine is also protected against RSV infection in a human challenge model [230]. Furthermore, coadministration of Ad26.RSV.preF with the Fluarix influenza vaccine in older adults showed an acceptable safety profile, with no observed interference in the immune response [231]. Of note, adding recombinant RSV preF protein may boost RSV‐specific humoral immunity. Studies have shown that the combined administration of Ad26.RSV.preF and RSV preF protein vaccines reduce RSV‐associated LRTDs in older adults [232]. Five, ChAd155‐RSV is an experimental chimpanzee adenovirus‐based RSV vaccine designed to express three proteins: F, N, and M2‐1. In a Phase I human randomized study, ChAd155‐RSV boosted specific humoral and cellular immunity with no major safety concerns [233]. In the phase I/II trial, ChAd155‐RSV was well tolerated in RSV‐seropositive children and induced neutralizing antibody titers against RSV‐A [234]. Moreover, ChAd155‐RSV administered to 6‐ to 7‐month olds showed a favorable safety profile and stimulated humoral immune responses [235]. However, the development of ChAd155‐RSV was discontinued as it was unlikely to achieve the target efficacy profile. Notably, a COVID‐19 adenovirus vector vaccine candidate identified new safety concerns associated with adenovirus vector vaccines, including vaccine‐induced immune thrombotic thrombocytopenia (VITT) [236]. Therefore, the development of adenovirus‐based RSV vaccines should prioritize the optimization of safety profiles, while ensuring robust and durable immunogenicity.

#### 7.2.3 Subunit Vaccines

Subunit vaccines are protein‐based and consist of purified fragments of the target pathogen. Since they lack the complete pathogen genome, they have enhanced safety [237]. The F glycoprotein on the surface of RSV facilitates viral entry into host cells and represents a critical target antigen for RSV vaccine development [238, 239]. Studies have found that the preF stabilized RSV F subunit vaccine plays a crucial role in eliciting potent neutralizing antibodies and memory B cells [138]. Currently, several subunit vaccine candidates are being developed for two target groups: pregnant women and older adults. DS‐Cav1 is a subunit vaccine stabilized in the preF conformation. According to Phase I clinical trial data, this subunit vaccine candidate induced higher serum‐neutralizing antibody levels, exhibited superior immunogenicity, and generated neutralizing antibody and B cell responses with greater functional characteristics [138, 240]. However, despite its excellent immunogenicity and high commercial potential, DS‐Cav1 rapidly loses its preF conformation when stored at 4°C, resulting in stalled commercial development. SCB‐1019 is the first bivalent RSV preF‐trimer subunit vaccine candidate in China, containing stabilized preF protein antigens targeting both RSV‐A and RSV‐B subtypes. Phase I clinical trial results showed that SCB‐1019 significantly increased neutralizing antibody titers against both RSV‐A and RSV‐B [241]. Other F protein‐based subunit vaccines developed independently in China are MKK900 (Maxvax Biotechnology, NCT06642558) and LYB005 (Guangzhou Patronus Biotechnology, NCT06442241), but there is limited information about them.

In addition to candidate vaccines using the F antigen, there are also candidate vaccines targeting non‐F antigens. DPX‐RSV(A) is a candidate vaccine targeting non‐F antigens. This vaccine, based on the extracellular domain (SHe) of the small hydrophobic (SH) protein from RSV subgroup A, is formulated with the oil‐based vaccine platform DepoVax [242]. A first‐in‐human study showed that DPX‐RSV(A) had high immunogenicity and acceptable safety, with sustained humoral responses observed for up to 180 days after vaccination [242]. Moreover, in a phase I clinical trial, DPX‐RSV induced SHe‐specific binding antibodies and T‐cell responses, supporting further clinical development [243]. Another candidate is the subunit vaccine VN‐0200. This candidate vaccine uses VAGA‐9001a as the antigen and MABH‐9002b as an immune stimulant. Currently, the results of the Phase I (NCT04914520) and Phase II (NCT05547087) clinical trials for VN‐0200 have not been publicly disclosed. Moreover, BARS13 is another subunit vaccine based on recombinant RSV G protein developed in China. It demonstrated good immunogenicity and safety in a first‐in‐human trial, inducing anti‐RSV‐G antibodies, and warranted further exploration in future clinical trials [244].

#### 7.2.4 Particle‐Based Vaccines

Particle‐based vaccines leverage virus‐like particle (VLP) or nanoparticle technology to present multiple antigens, activating robust humoral and cellular immune responses, thereby providing an innovative platform for RSV vaccine development. The RSV F nanoparticle‐based vaccine is being assessed for three target groups: (1) infants via maternal vaccination, (2) children aged 6 months to 5 years, and (3) adults aged 60 and older [218]. SynGEM is an RSV candidate vaccine containing F protein linked to a bacterium‐like‐particle (BLP) carrier. In this vaccine platform, antigens are presented by the bacterial particles. Phase I clinical trial showed that SynGEM induced sustained antibody response in human volunteers [245]. V‐306 is a self‐assembling VLP candidate vaccine existing with multiple epitopes of antigenic site II of the RSV F protein (FsII) on its surface. V‐306 has been evaluated for safety and immunogenicity in cotton rats, mice, and rabbits. The vaccine candidate exhibited strong immunogenicity in both mice and rabbits and provided complete protection against vaccine‐associated enhanced respiratory disease (VAERD) in mouse and cotton rat challenge models. Furthermore, in a Phase I clinical trial, V‐306 demonstrated a favorable safety profile. An increasing trend in epitope‐specific FsII‐IgG antibodies was observed in the medium‐ and high‐dose groups, although the increase diminished following the second dose [246]. IVX‐121 is a VLP candidate vaccine based on the RSV preF protein. Preclinical studies showed that IVX‐121 induced a neutralizing antibody response 10 times higher than that of DS‐Cav1 alone. The Phase I clinical trial demonstrated that the neutralizing antibody response to RSV remained highly persistent for up to 12 months following a single administration of IVX‐121. One month after revaccination, IVX‐121 elicited a robust RSV‐A immune response, with no observed increase in RSV‐B titers [247].

#### 7.2.5 Chimeric Vaccines

Chimeric vaccines are based on attenuated viruses from related pathogens, engineered to express specific genes of the target virus. They can enhance antigen presentation, effectively activating an adaptive immune response [248]. Two chimeric vaccines currently in development for RSV are rBCG‐N‐hRSV and SeV/RSV [215]. rBCG‐N‐hRSV is a recombinant *Mycobacterium bovis* bacillus calmette guerin (BCG) vaccine that expresses the human RSV N. Phase I clinical trial showed that the rBCG‐N‐hRSV vaccine induced specific cellular and humoral responses [249]. SeV/RSV is a replication‐competent vaccine based on the Sendai virus (SeV) that carries the RSV F protein gene. In Phase I clinical trials, the vaccine demonstrated a favorable safety profile and robust immunogenicity [250]. Both vaccines have been proven to have good safety profiles.

#### 7.2.6 Nucleic Acid Vaccines

mRNA vaccines offer high antigen expression efficiency, strong safety, and robust immunogenicity. They do not pose the risk of nucleic acid integration into the host genome and can naturally degrade in vivo, while simultaneously activating both humoral and cellular immunity, making them highly promising for widespread application [251, 252]. With the tremendous success of mRNA vaccines in combating COVID‐19, mRNA vaccines have undergone rapid development, and several RSV mRNA vaccines have entered clinical trials. Current RSV vaccine candidates focus on the highly conserved F protein, as targeting the preF conformation elicits a strong neutralizing antibody response [253].

IN006 is a bivalent RSV mRNA vaccine independently developed in China. This candidate vaccine contains mRNA encoding the stabilized preF proteins of RSV‐A and RSV‐B, targeting both viral subgroups simultaneously. Preclinical studies showed that IN006 had a favorable safety profile, exhibited robust humoral and cellular immune activation capabilities, and provided effective protection against both RSV‐A and RSV‐B in cotton rat challenge models. It is currently undergoing Phase I clinical trials (NCT06645665, NCT06287450). Another self‐developed mRNA vaccine candidate, STR‐V003, encapsulates modified mRNA encoding the RSV preF protein within lipid nanoparticles (LNPs). This vaccine has shown strong immunogenicity in both mice and cotton rats, effectively inducing high levels of neutralizing antibodies and RSV preF‐specific IgG antibodies. It also significantly reduced RSV viral loads in the lungs and nasal tissues of challenged animals. As a result, STR‐V003 has been granted FDA approval to advance to a Phase I clinical trial (NCT06344975).

mRNA‐1777 is also an investigational prophylactic RSV vaccine encoding the full‐length RSV F protein stabilized in the prefusion conformation. The phase I trial assessed the safety, tolerability, and immunogenicity of the mRNA‐1777 vaccine [254]. Results indicated that mRNA‐1777 enhanced RSV F‐specific humoral and cellular immunity and was well tolerated [254]. Notably, mRNA‐1345 (mRESVIA) is the first marketed RSV mRNA vaccine. Phase I clinical trials indicated that the mRNA‐1345 vaccine demonstrated an acceptable safety profile and strong immunogenicity, inducing a durable antibody response [255, 256]. In phase III clinical trials, a single dose of the mRNA‐1345 vaccine enhanced RSV‐A and RSV‐B neutralizing antibody titers, and RSV preF‐binding antibody concentrations, as well as resulted in no evident safety concerns and led to a lower incidence of RSV‐associated LRTDs and of RSV‐associated acute respiratory disease than placebo among adults 60 years of age or older [257, 258]. The development of these vaccines highlights the transformative potential of mRNA technology in RSV vaccine development.

### 7.3 RSV Mucosal Vaccines

However, even more research suggests that an ideal RSV vaccine should induce both systemic and mucosal immune responses to protect the upper and lower respiratory tracts [259]. Importantly, numerous studies have shown that RSV‐specific mucosal antibody levels correlate more strongly with protection against RSV infection than serum antibody titers [137, 260, 261]. For example, a clinical study demonstrated that high levels of RSV‐specific mucosal IgG correlated more strongly with reduced viral load and inflammation compared to plasma IgG levels [260]. These findings suggest that mucosal vaccination, designed to induce a robust mucosal immune response, may represent a more effective vaccination strategy. Mucosal immunity has been shown to effectively induce secretory IgA and T_RM_ cell responses, offering the potential for preventing viral infections and transmission [262]. Currently, extensive research is also focused on developing RSV mucosal vaccines. SynGEM is a novel intranasal subunit vaccine composed of empty BLP linked to the RSV F glycoprotein. SynGEM has been shown to be safe in healthy adults and capable of inducing sustained local and systemic antibody responses [245]. BLB‐201 is a live viral vector intranasal RSV candidate vaccine. In a phase I clinical study, BLB‐201 was shown to elicit cellular, humoral, and mucosal immune responses [227]. Additionally, several LAVs have been assessed for their immunogenicity via intranasal administration. For instance, RSV/ΔNS2/Δ1313/I1314L, attenuated by the deletion of the NS2 gene and the introduction of a temperature‐sensitive mutation in the polymerase gene, demonstrated both immunogenicity and genetic stability in RSV‐seronegative children aged 6–24 months. This vaccine effectively increased serum RSV‐neutralizing antibody titers and anti‐RSV F IgG levels in recipients a [221]. Similarly, LID/ΔM2‐2/1030s, which lacks the RSV M2‐2 regulatory protein and contains a stabilized temperature‐sensitive 1030s mutation in the polymerase, was shown to induce durable immunity in RSV‐seronegative children [263]. Another live‐attenuated RSV vaccine, LIDΔM2‐2, attenuated through the deletion of the RNA regulatory protein M2‐2, exhibited excellent immunogenicity and significantly elevated serum‐neutralizing antibody levels in RSV‐seronegative children [264]. RSV/6120/ΔNS2/1030s is a cDNA‐derived live‐vaccine candidate attenuated by deletion of the interferon antagonist NS2 gene and the genetically stabilized 1030s missense polymerase mutation in the polymerase, conferring temperature sensitivity. Phase I clinical trials showed that RSV/6120/ΔNS2/1030s infected 100% of RSV‐seronegative vaccinees and was immunogenic and genetically stable [265]. These findings highlight the potential of RSV vaccines administered via intranasal immunization to provide robust and durable protection. Meanwhile, these vaccine candidates represent promising strategies for preventing RSV infections in vulnerable groups, such as young children.

## 8 Perspectives and Conclusions

RSV, as a pathogen that poses a significant threat to the health of infants, the elderly, and immunocompromised individuals, has seen remarkable advancements in both basic research and clinical interventions in recent years. Studies on the pathophysiology of RSV, particularly the roles of nonstructural proteins NS1 and NS2 in interfering with the host interferon response, as well as the high immunogenicity exhibited by the RSV F protein in its preF conformation, have provided critical targets for vaccine design and antiviral drug development. Meanwhile, immunological studies focusing on RSV‐specific mucosal and systemic immunity have further clarified the relationships between different antibody types, immune sites, and their mechanisms of action. These findings not only help explain the infection and transmission characteristics of RSV but also provide important theoretical guidance for optimizing vaccine immunization strategies.

In the field of vaccine development, various candidate vaccines, including live‐attenuated vaccines (such as RSV/ΔNS2/Δ1313/I1314L and LID/ΔM2‐2), subunit vaccines (such as BARS13 and RSVpreF), adenoviral‐vector vaccines (such as Ad26.RSV.preF), mRNA vaccines (such as mRNA‐1345), and mucosal vaccines (such as SynGEM and BLB201), have shown promising prospects in different populations and clinical stages. These vaccines, through innovative designs such as antigen modifications based on the RSV preF protein, temperature‐sensitive mutations, and mucosal immunity induction strategies, have significantly enhanced immunogenicity and safety. Some of these vaccines have already been approved or are nearing market approval. Furthermore, research on mucosal vaccines has highlighted the importance of inducing local immunity, particularly for upper respiratory tract protection, offering a new perspective for future immunization strategies. Significant progress has also been made in the field of RSV treatment. The development of novel neutralizing antibodies, such as nirsevimab (MEDI‐8897), offers robust protection for high‐risk populations through passive immunity, while antiviral drugs targeting the RSV polymerase are providing more options for postinfection treatment. However, the comprehensive advancement of RSV prevention and treatment still faces several challenges, including individual variations in immune responses, accessibility of vaccines and antibodies, and the potential impact of viral mutations on the effectiveness of vaccines and therapies.

Future RSV research should focus on several key areas. First, leveraging multidisciplinary collaboration to delve deeper into the immunological and molecular mechanisms of RSV infection, particularly the interactions between the virus and its host. Second, optimizing immunization strategies tailored to the specific needs of different populations, such as newborns, the elderly, and pregnant women. Third, exploring the potential of combination vaccines and multi‐targeted interventions while advancing research on mucosal immunity to provide more comprehensive protection. In summary, RSV research has gradually matured, encompassing fundamental mechanisms, vaccine development, and clinical applications. Continued technological breakthroughs and cross‐disciplinary cooperation will further drive the prevention and control of this globally significant pathogen, bringing profound and positive impacts on human health.

## Author Contributions

Jie Shi, Xiya Huang, and Xiawei Wei designed the study and drafted the original manuscript. Chunjun Ye, Yishan Lu and Yanyan Liu drew figures and provided some important guidance on the revised manuscript. Xiawei Wei and Yuquan Wei were involved in the critical revision of the manuscript and provided financial support and final approval of the version. All authors have read and agreed to the published version of the manuscript.

## Conflicts of Interest

The authors declare no conflicts of interest.

## Supporting information

Supplementary Information.

## Data Availability

The authors have nothing to report.
