# Supplementary material for: Respiratory Syncytial Virus (RSV): A Comprehensive Overview From Basic Biology to Clinical Prevention and Control
Source: Med Res Rev. 2025 Nov 19;46(3):672–712. doi: 10.1002/med.70025 (PMC13058691; doi:10.1002/med.70025)
Supplement: Supplementary file 1 — Supplementary Information. [file MED-46-672-s001.docx]

Please complete the following regarding your submission and upload this file at the time of submission as “Supplementary Information – Not for Review.”

1. **Title:**

Respiratory Syncytial Virus (RSV): A Comprehensive Overview from Basic Biology to Clinical Prevention and Control

1. **Author(s):**

Jie Shi^1,#^, Xiya Huang^1,#^, Chunjun Ye^1,#^, Yishan Lu^1^, Yanyan Liu^1^, Yuquan Wei^1*^, Xiawei Wei^1*^

1. **A link to the corresponding author’s published papers, such as ORCID or lab website:**

Xiawei Wei：<https://orcid.org/0000-0002-6513-6422>

Yuquan Wei：<https://orcid.org/0000-0002-6211-3217>

1. **Keywords, separated by a semicolon:**

RSV; basic biology; clinical prevention; clinical control; RSV vaccines.

1. **Brief justification for this review:** *Why is this particular review timely and distinct from other reviews in the field?*

In recent years, respiratory syncytial virus (RSV) has demonstrated new epidemiological patterns globally, particularly following the COVID-19 pandemic, with notable shifts in seasonality and transmission dynamics. Concurrently, major breakthroughs in RSV vaccine development and long-acting monoclonal antibodies have been achieved, with several candidates completing pivotal clinical trials and receiving regulatory approval—signaling a new era in RSV prevention. At this critical juncture, a comprehensive review synthesizing the latest advances in RSV molecular biology, immune evasion mechanisms, epidemiological trends, and clinical interventions is both timely and of significant theoretical value. Unlike existing reviews that tend to focus on isolated aspects such as pediatric infection or vaccine development, this review aims to construct an integrated framework encompassing virology, host immunity, population-level transmission, and therapeutic strategies, offering a broad scientific foundation for future basic and translational research.

1. **Brief summary (~5 sentences) of the conclusion / take-home message of your manuscript:**

This review provides a comprehensive summary of the latest research developments on RSV, including its basic biological characteristics, epidemiology, clinical manifestations, diagnostic methods, treatment strategies, and long-term health impacts. In addition, we highlight recent breakthroughs in understanding immune response mechanisms, as well as advances in vaccine development and other preventive measures. Through this work, we aim to offer clinicians, researchers, and public health professionals an in-depth and forward-looking scientific foundation for understanding RSV and guiding future prevention strategies.

1. **Years of literature covered?** (i.e., 2013-present)

1994- present

1. **List of the most related publications by the author(s**)**:**

Lei H, Alu A, Yang J, He C, Shi J, Hong W, Peng D, Zhang Y, Liu J, Qin F, Huang X, Ye C, Pei L, He X, Yan H, Lu G, Song X, Wei X, Wei Y. Intranasal Inoculation of Cationic Crosslinked Carbon Dots-Adjuvanted Respiratory Syncytial Virus F Subunit Vaccine Elicits Mucosal and Systemic Humoral and Cellular Immunity. MedComm (2020). 2025 Mar 24;6(4):e70146. doi: 10.1002/mco2.70146. PMID: 40135196; PMCID: PMC11933438.
